# Fostering the Dense Packing of Halide Perovskite Quantum Dots through Binary-Disperse Mixing

**DOI:** 10.1021/acsnano.3c07688

**Published:** 2023-10-03

**Authors:** Shiang Li, Ziqi Wang, Yuhao Li, Chun-Jen Su, Yuang Fu, Yi Wang, Xinhui Lu

**Affiliations:** † Department of Physics, The Chinese University of Hong Kong, Hong Kong SAR 999077, China; ‡ Spallation Neutron Source Science Center, Institute of High Energy Physics, Chinese Academy of Sciences, Dongguan 523803, China; § National Synchrotron Radiation Research Center, Hsinchu Science Park, Hsinchu 30076, Taiwan

**Keywords:** quantum dots, binary size, packing, GISAXS, solar cells

## Abstract

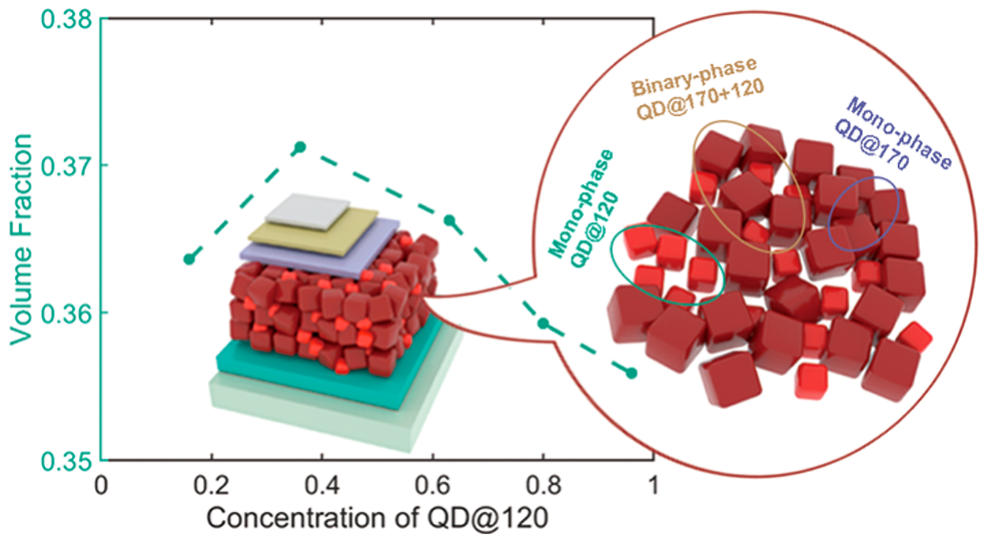

Due to their versatile applications, perovskite quantum dot (PQD)-based optoelectrical devices have garnered significant research attention. However, the fundamental packing behavior of PQDs in thin films and its impact on the device performance remain relatively unexplored. Drawing inspiration from theoretical models concerning packing density with size mixtures, this study presents an effective strategy, namely, binary-disperse mixing, aimed at enhancing the packing density of PQD films. Comprehensive grazing-incidence small-angle X-ray characterization suggested that the PQD film consists of three phases: two monosize phases and one binary mixing phase. The volume fraction and population of the binary-size phase can be tuned by mixing an appropriate amount of large and small PQDs. Furthermore, we performed multi-length-scale all-atom and coarse-grained molecular dynamics simulations to elucidate the distribution and conformation of organic surface ligands, highlighting their influence on PQD packing. Notably, the mixing of two PQDs of different sizes promotes closer face-to-face contact. The densely packed binary-disperse film exhibited largely suppressed trap-assisted recombination, much longer carrier lifetime, and thereby improved power conversion efficiency. Hence, this study provides fundamental understanding of the packing mechanism of perovskite quantum dots and highlights the significance of packing density for PQD-based solar cells.

First synthesized by Protesescu et al. in 2015,^1^ all-inorganic CsPbI_3_ perovskite quantum dots (PQDs) have demonstrated higher luminescence and more stable black phase at room temperature than their bulk counterparts.^2,3^ Nowadays, CsPbI_3_ PQDs have been widely applied in versatile optoelectronic devices such as solar cells (SCs), light-emitting diodes (LEDs), lasers, and photodetectors,^4−8^ owing to their properties including size-dependent tunability in absorption and emission bands^4^ as well as energy levels,^9^ heavy doping capability,^10,11^ multiple exciton effects,^12,13^ and layer-by-layer processability.^14,15^ The first CsPbI_3_ PQD based solar cell was fabricated by Luther and co-workers, who achieved a power conversion efficiency (PCE) of over 10%.^4^ To date, the PCE of CsPbI_3_ PQD solar cells has been improved through composition engineering,^16,17^ surface engineering,^18,19^ and energy band engineering.^9,14^ Their performance, however, still lags far behind that achieved by bulk perovskites.^20−22^

The major challenge lies in the impeded charge transport between PQDs, because of the insulating nature of long organic capping ligands, such as oleic acid (OA) and oleylamine (OAm), which are introduced during the synthesis process to terminate PQD surfaces and control their size. Therefore, it is desirable to shorten the interparticle distance between PQDs to establish stronger tunnel coupling. For instance, the decrease of interparticle distance from 5.8 to 3.7 nm was reported to lower the charge transfer rate from ∼150 ns^–1^ to ∼2 ns^–1^ for PbS QDs.^23^ Replacing the long-chain ligands with short-chain ligands is one common solution.^24,25^ In general, the long chain OA ligands can be replaced by short-chain acidic anionic ligands, such as acetate (OAc^–^), while the long chain OAm ligands can be substituted by short-chain basic cationic ligands, such as formamidium (FA^+^) and guanidinium (GA^+^). However, ligand exchange is usually conducted as a postsynthesis treatment on the long chain terminated PQD film, at which point the packing motif of PQDs is largely determined during the film deposition process. Thus, ligand exchange is not effective at eliminating the voids caused by the loose packing of PQDs. The fundamental packing behavior of PQDs in thin films and the impact on the device performance are yet to be explored.

In this work, we report an effective approach applying a binary-disperse mixing of QDs to increase the packing density of spin-coated CsPbI_3_ PQD films and enhance their solar cell device performance. The theoretical model for describing the packing density with arbitrary sphere mixture was proposed by Farr and Groot in 2009.^26^ They predicted that binary mixing could lead to an increase in volume fraction compared with the theoretical volume fraction limit for monodisperse spherical particles at random close packing. Therefore, we systematically investigated the packing status of binary-disperse PQD films with the help of grazing-incidence small-angle X-ray scattering (GISAXS). Our fitting results revealed that a binary-disperse PQD film consisted of three phases: two monosize phases and one binary-mixing phase. The maximum volume fraction of 37.1% was achieved at a number ratio of 0.64 (14 nm)/0.36 (10 nm). Molecular dynamics simulations indicated the significant influence of organic surface ligands on the packing of PQDs, with binary mixing leading to enhanced face–face contact. Interestingly, the densely packed binary-disperse film showcased substantial suppression of trap-assisted recombination, resulting in an extended carrier lifetime, more efficient charge transport, and consequently, an improved power conversion efficiency of 14.42% with a *J*_SC_ of 17.08 mA cm^–2^, a *V*_OC_ of 1.19 V, and a fill factor of 71.12%. This work demonstrates the importance of packing density for PQD-based solar cells and provides a straightforward recipe to effectively enhance the packing density and, in turn, the device performance.

## Results

### The Synthesis of CsPbI_3_ QDs with Two Different Sizes

**Figure 1:**
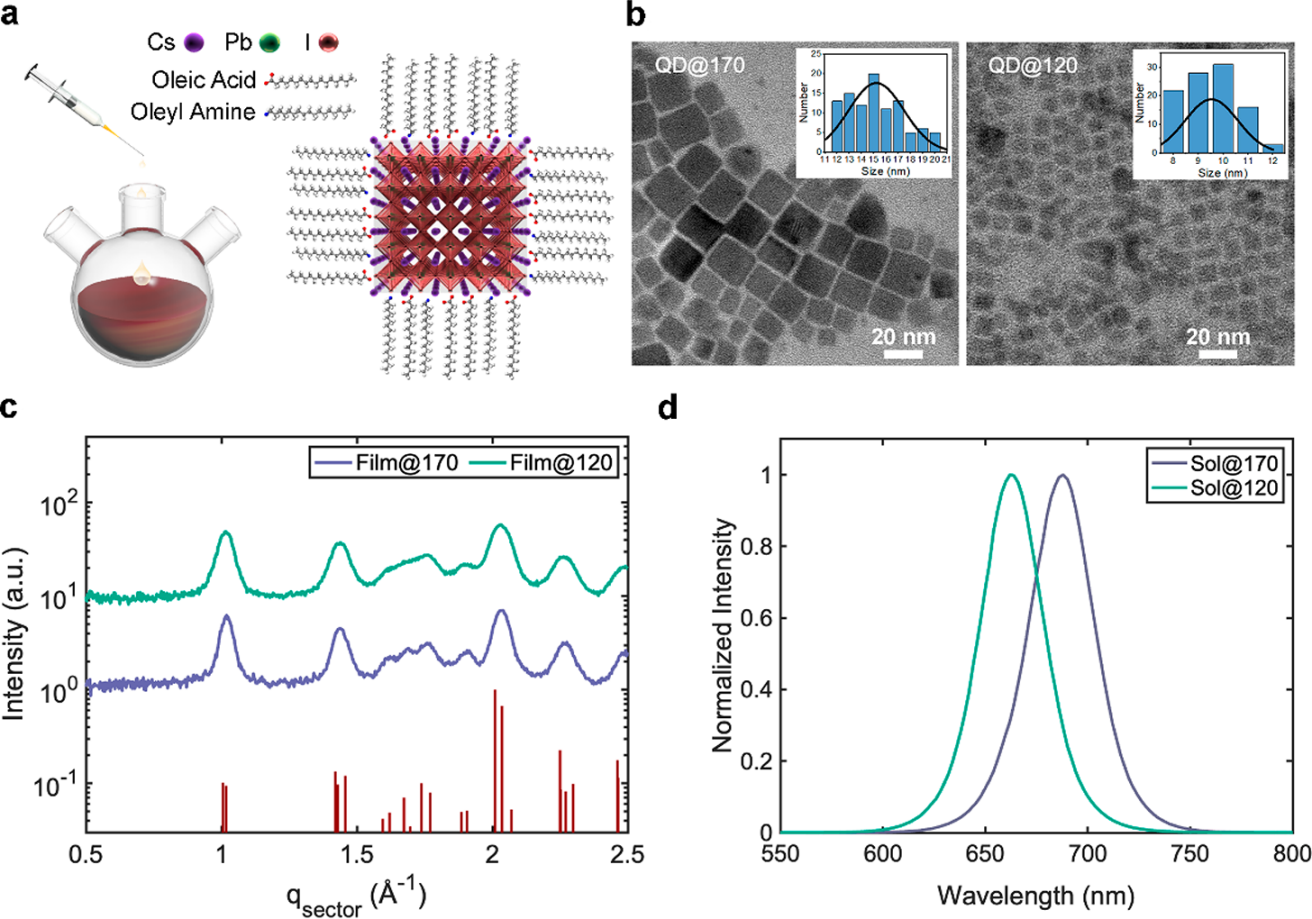
Illustration of synthesis and basic characterization of PQDs. (a) Illustration of synthesis via the hot injection method (left) and molecular structure with ligands attached on the surface (right). (b) TEM images of synthesized QDs. Inset: size distribution statistics. (c) Film GIWAXS sector intensity profiles of PQDs synthesized at 170 and 120 °C. (d) Solution PL spectra of PQDs synthesized at 170 and 120 °C.

CsPbI_3_ PQDs were synthesized via a hot injection method as illustrated in Figure 1a.^27^ Briefly, PbI_2_ powder was dissolved in 1-octadecene (ODE) together with oleic acid (OA) and oleylamine (OAm) to form a precursor solution. Then, the precursor was degassed at 120 °C for 1 h to remove the moisture and oxygen, followed by the injection of CsAc precursor predissolved in OA at two different temperatures of 170 and 120 °C in order to grow QDs of two different sizes. Therefore, the synthesized PQDs were named QD@170, QD@120, respectively. Detailed post-treatment processes can be found in the Methods section. TEM images are shown in Figure 1b, suggesting that the PQDs are generally cubic in shape, consistent with previous reports.^27,28^ Statistical analysis indicates that the average size of QD@170 is around 14 nm, while that of QD@120 is around 10 nm. Figure 1c shows the sector intensity profiles versus wavenumber *q* of two-dimensional (2D) grazing-incidence wide-angle X-ray scattering (GIWAXS) patterns measured from the films made of pure QD@120 and pure QD@170, respectively (2D images are shown in Figure S1). The positions of characteristic diffraction peaks indicate that both PQDs are in the same crystalline γ-phase.^28,29^ Normalized photoluminescent (PL) emission spectra are displayed in Figure 1d. QD@170 and QD@120 exhibit emission peaks at 688 and 662 nm, respectively, in agreement with previously reported results for γ-CsPbI_3_ QDs with similar sizes.^4^

### The Packing of Binary-Disperse CsPbI_3_ PQDs in Thin Films

**Figure 2:**
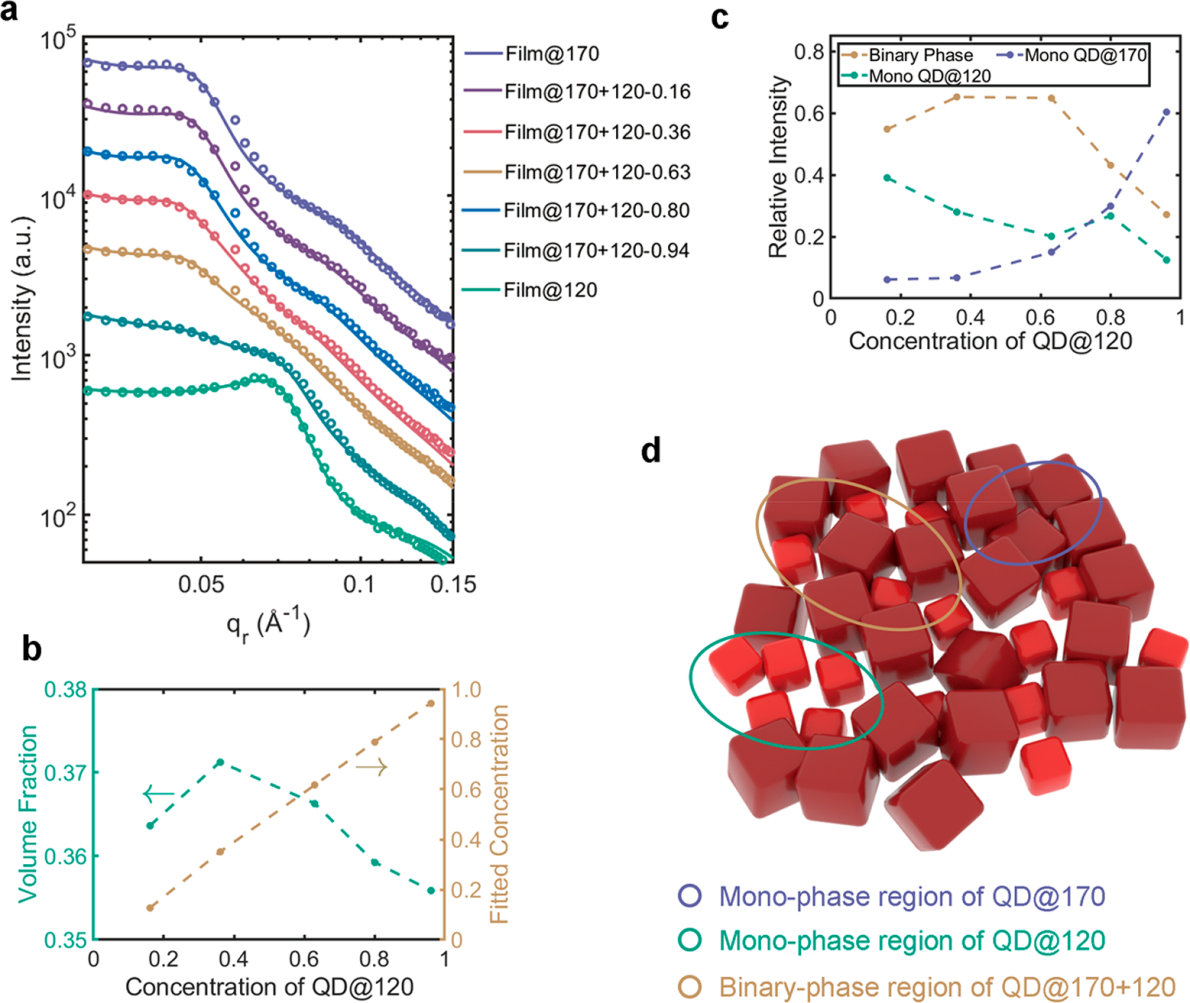
GISAXS measurement and schematic packing motif. (a) GISAXS profiles (circles) and fitting results (solid lines) of pure QD@170 and QD@120 films and binary-disperse QD film. The numbers after Film@170+120 in the legend denote the feed concentrations of QD@120. (b) Fitted volume fraction and concentration of QD@120 in the binary-mixing phase versus feed concentration of QD@120 and (c) corresponding scattering intensity contribution of each phase. (d) Schematic of the three phases in a binary-disperse QD film.

Next, QD@170 and QD@120 were mixed to form binary-disperse CsPbI_3_ PQD films. The number ratio of a given mixture was calculated by the weight ratio divided by the size ratios of QD@170 and QD@120. All the films were formed by spin-coating precursor solutions of 70 mg/mL at 1000 rpm for 10 s, followed by a faster spinning at 2000 rpm for 7 s, the same as the fabrication procedure of monodisperse PQD films. To understand the packing motif of CsPbI_3_ PQDs in thin films, grazing-incidence small-angle X-ray scattering (GISAXS) measurements were carried out (Figure 2a and Figure S2). Figure 2a presents horizontal line cuts of two-dimensional (2D) GISAXS patterns (Figure S2) at the Yoneda peak position.^30,31^ As illustrated in Figure 2d, the overall scattering intensity arises from contributions by three types of phases: pure QD@170 phase, pure QD@120 phase, and binary-mixing phase, in the form:
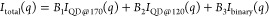
1For the pure QD phase, the intensity contribution follows the formula
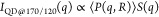
2where ⟨*P*(*q*, *R*)⟩ is the spherical form factor with an average radius of *R* following Schultz distribution^30,31^ and *S*(*q*) is the hard-sphere structure factor with Percus–Yevick (P–Y) approximation.^32^

For the binary mixing phase, the intensity profile follows the relation^33^
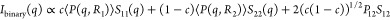
3where *c* is the concentration of QD1, ⟨*P*(*q*, *R*_*i*_)⟩ is the form factor the same as that of pure QD phase, *P*_12_ is estimated by , and *S*_*ij*_ is the structure factor calculated from the Fourier transforms of correlation functions *C*_*ij*_ with P–Y approximation.^33^ Detailed formulae can be found in the Supporting Information, Theoretical Modeling.

The intensity profiles of pure QD@170 and pure QD@120 films were fitted with eq 2, and the average sizes (2*R*) are 12.6 and 8.7 nm, respectively, consistent with the TEM results. The packing volume fraction of QD@120 is found to be 35.7%, larger than that of QD@170 (34.7%). When more QD@120 are added to pure QD@170 system, the scattering profiles gradually evolve to the scattering profile of pure QD@120. We fitted these curves with eq 1, and the fitting results are summarized in Table 1 and Table S1. As shown in Figure 2b, the fitted concentration of QD@120 agrees with the experimental number ratio used to prepare the binary-disperse film. Intriguingly, when more and more QD@120 is added to the pure QD@170 system, the packing volume fraction gradually increases and peaks at the number ratio of QD@170/QD@120 = 0.64:0.36 (denotes as Film@170+120-0.36). The relative scattering contribution of each phase at different number ratios is then calculated via  and plotted in Figure 2c. It is evident that binary-mixing phases contribute dominantly when the number ratio is far from 1:0 or 0:1, demonstrating that the overall volume fraction can be effectively increased by binary-disperse mixing. Regarding the reason there are phase separations, we think it should refer to the classical Flory–Huggins theory,^34^ where the mixing of two polymers naturally separates into two pure polymer phases and one two-component intermixing phase, while the intermixing status is determined by many factors, including monomer interaction, temperature, and composition. Analogous to polymers, the mixing of the two-sizes of PQDs may also lead to the formation of three phases–two monosized phases and one binary-mixing phase.^34^ The intermixing degree should depend on the interaction between the two sizes of PQDs in terms of different surface energy as well as ligand densities and distributions.^10,35^

**Table 1:** Fitted Concentrations and Volume Fractions of QD@120 in Various Feed-Concentration QD films

feed concentration of QD@120	0	0.16	0.36	0.63	0.80	0.94	1
fitted concentration of QD@120	0	0.13	0.35	0.62	0.79	0.94	1
fitted volume fraction, η (%)	34.7	36.4	37.1	36.6	35.9	35.6	35.7

### Computer Modeling of the Influence of Organic Ligands on PQD Packing

While the trend of binary-component volume fraction shown in Table 1 agrees with the theoretical prediction on binary mixing,^26^ the fitted volume fraction values are much lower than those predicted for random close packing. This may be largely due to the negligible scattering contribution of organic ligands, as their electron density is much lower than that of the CsPbI_3_ interior part. In the meantime, it suggests that simply modeling the CsPbI_3_ PQDs as hard spheres may not be sufficient to characterize their packing, during which organic ligands can play a non-negligible role. Therefore, we next performed multi-length-scale all-atom and coarse-grained molecular dynamics (MD) simulations to examine the distribution and conformation of organic ligands on the PQD surface and probe their influence on PQD packing.

**Figure 3:**
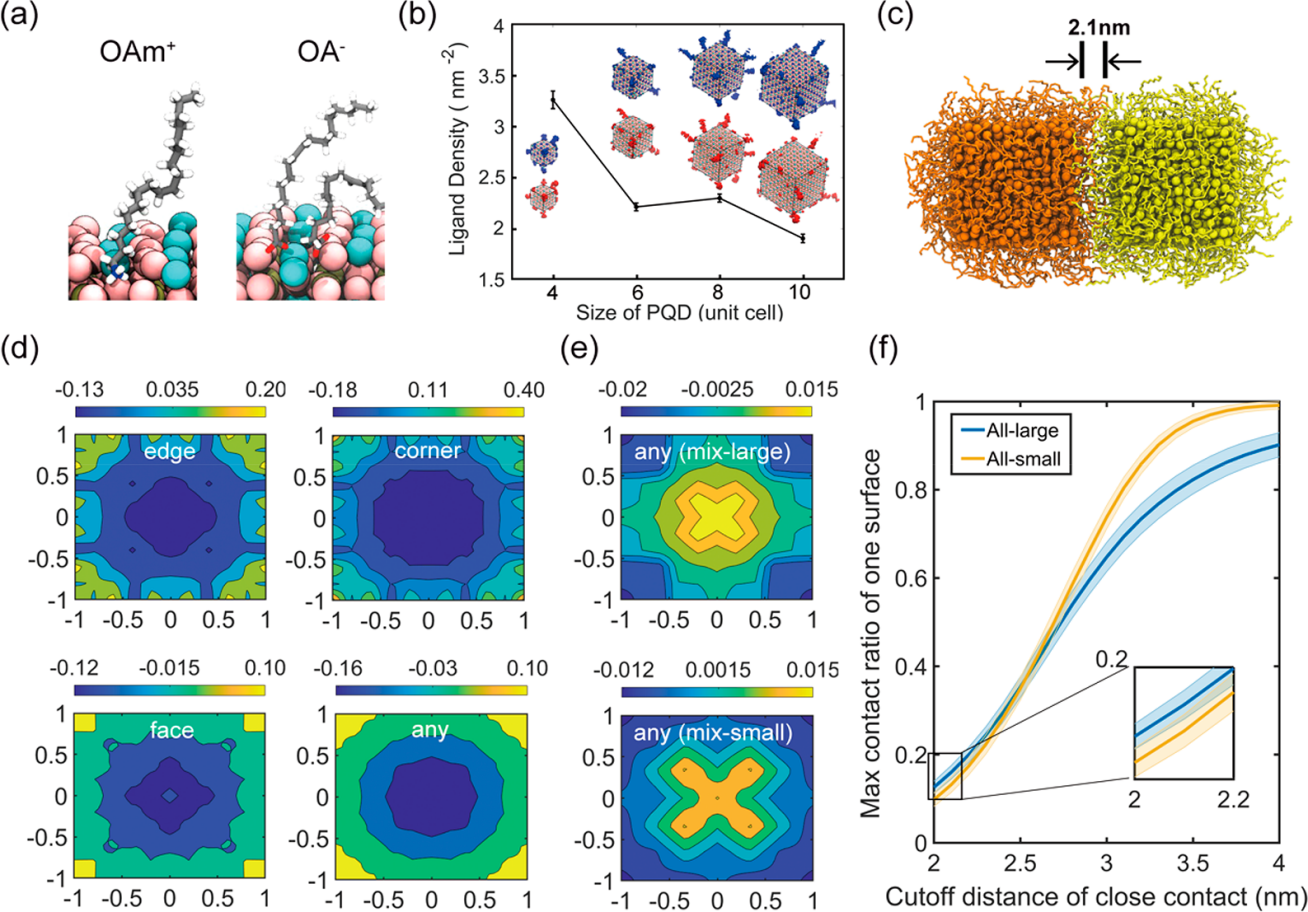
MD simulation of PQD and contact frequency map. (a) Representative conformations of OAm^+^/OA^–^ attached to the surface of a PQD (Cs, cyan; Pb, yellow; I, pink; N, blue; O, red; C, gray; H, white). (b) Density of long-chain ligands on PQDs. Inset shows the 3D occupancy isosurfaces (5%) of the OA^–^ (red) and OAm^+^ molecules (blue), reflecting the preferential binding of these ligands on the edges and corners of a PQD. (c) Simulation snapshot of two PQDs with restrained surface-to-surface orientation. (d) Contact difference maps (all-large subtracting all-small) computed for a selected atom on a given PQD (coordinates mapped onto the [−1, 1] by [−1, 1] square) and the edge/corner/face/any atom of a second PQD (see Supporting Information MD simulation of details). Positive and negative values indicate regions with higher and lower contact frequency, respectively, in the all-large system relative to the all-small system. (e) Contact difference maps computed for the 1:1-mix system subtracting the all-large or all-small system. (f) Maximum contact ratio among the six surfaces of a PQD computed from coarse-grained simulations of the all-large (blue) and all-small (yellow) systems.

First, all-atom MD simulations were employed to examine the conformation and distribution of ligands on the passivated PQD surface. Terminated with Cs–I-rich (100) planes, the PQD surfaces were first preinserted with the cationic OAm^+^ (see Supporting Information, MD simulation, for details). The system was then solvated in octane as well as additional free OAm^+^ and OA^–^. A typical simulation box is shown in Figure S3. Throughout multiple 100 ns production runs, preinserted OAm^+^ ligands were found to be intriguingly stable on the PQD surfaces, consistent with earlier modeling results of perovskite nanocrystals passivated with alkylammonium ligands.^36^ In addition to these preinserted ligands, passivation of PQD was further achieved by surface attachment of free OAm^+^ and OA^–^ originally placed in the octane solution. The vast majority of the ligands attached onto a PQD directed their charged heads toward it (Figure 3a), reflecting the important role played by electrostatics in ligand–PQD association. As shown in Figure S3, the preinserted OAm^+^ as well as newly attached OAm^+^ and OA^–^ formed a dense layer around a PQD, resulting in a ligand density in the range of 1.9 to 3.2 nm^–2^ for the four PQD models simulated (Figure 3b). Further analysis of ligand distribution reveals that apart from preinserted ligands, both OAm^+^ and OA^–^ preferentially bind at the edges and corners of a PQD, as reflected by their 3D occupancy isosurfaces shown in illustrations inserted in Figure 3b. A similar distribution was also observed in PQDs passivated with the short-chain ligands FA^+^/OAc^–^ (Figure S4b). Such uneven ligand distributions likely arise from the strong electrostatic potential near the edge and corner regions of the PQD cubes (Figure S5). Since the percentage of surface atoms residing on the edges and corners decreases as the size of a PQD increases, the above preferential binding of ligands explains the drop in ligand density of larger PQDs (Figure 3b), consistent with the trend of ligand density (Table S2) calculated from experimental results of ICP-OES (Figure S6) and NMR (Figure S7). Next, we performed all-atom MD simulations to further examine how closely two PQDs could approach each other when all free ligands in solution (excluding those preinserted and attached onto PQD surfaces) and the octane solvent were removed and the two PQDs were restrained such that they could only approach each other in the orientation shown in Figure 3c. The two PQDs were allowed to adjust their distance freely and the average surface-to-surface distance between them was measured to be 2.1 nm, reflecting the interpenetration of ligands from the neighboring PQDs, since the free extended ligand shell of each PQD reached approximately 2 nm (see Supporting Information, Softness, for detail). Based on these results, a crude estimation of the volume fractions of PQDs with long-chain ligands included can be made: with the average thickness of the ligand shell taken into account (Figure S8), the volume fraction rises to 55.1% and 68.4% for film of QD@170 and film of QD@120, respectively, comparable to the limit of random packing.^37^

Next, to further probe the interactions between PQDs after spin coating and prior to ligand exchange, we constructed coarse-grained (CG) models of large (10.5-unit cell) and small (6.5-unit cell) PQDs with preinserted and attached long-chain ligands and mixed them at three number ratios (large/small = 1:0, 1:1, 0:1, named as all-large, 1:1-mix, and all-small systems, respectively). Through altogether ∼5 μs CG simulations, we first computed the minimum distance between any two PQDs, which is found to peak at smaller values as the number of large PQDs increases (Figure S9), consistent with their decreased ligand density. This metric, however, reflects only the distance between the two closest atoms from neighboring PQD pairs. To further characterize the contact patterns of all surface atoms on a given PQD, we then determined the contact frequency between a selected atom on a given PQD and the edge, corner, face, or any of the above atoms on a second PQD (see Supporting Information, MD simulation, for details). The contact difference maps shown in Figure 3d, obtained by subtracting the contact maps of the all-small system from those of the all-large system, collectively reflect the increased corner and edge contact of the large PQD relative to the small one. Conversely, the small PQD establishes more face-to-face contact (Figure 3d) and exhibits a larger contact ratio for its best packed surface than the large PQD (Figure 3f). These differences may have arisen from the different ligand densities and sizes of the two PQDs, where the higher ligand density of the small PQD serves to improve its “cushioning”, enabling the small PQD to better adapt to various geometry requirements encountered during packing and rendering a more uniform contact map for its surface atoms than the large PQD with a lower ligand density (Figure S10). Interestingly, once a monodisperse system is incorporated with a second PQD of a different size, an increased level of contact is seen at the face of the PQD, accompanied by decreased corner and edge contact (Figure 3e). These changes reflect a more favorable face–face contact upon the mixing of two PQDs of different sizes, in line with the experimentally observed higher packing density of the binary-disperse systems.

### Packing Density-Dependent Device Performance and Trap Density

**Figure 4:**
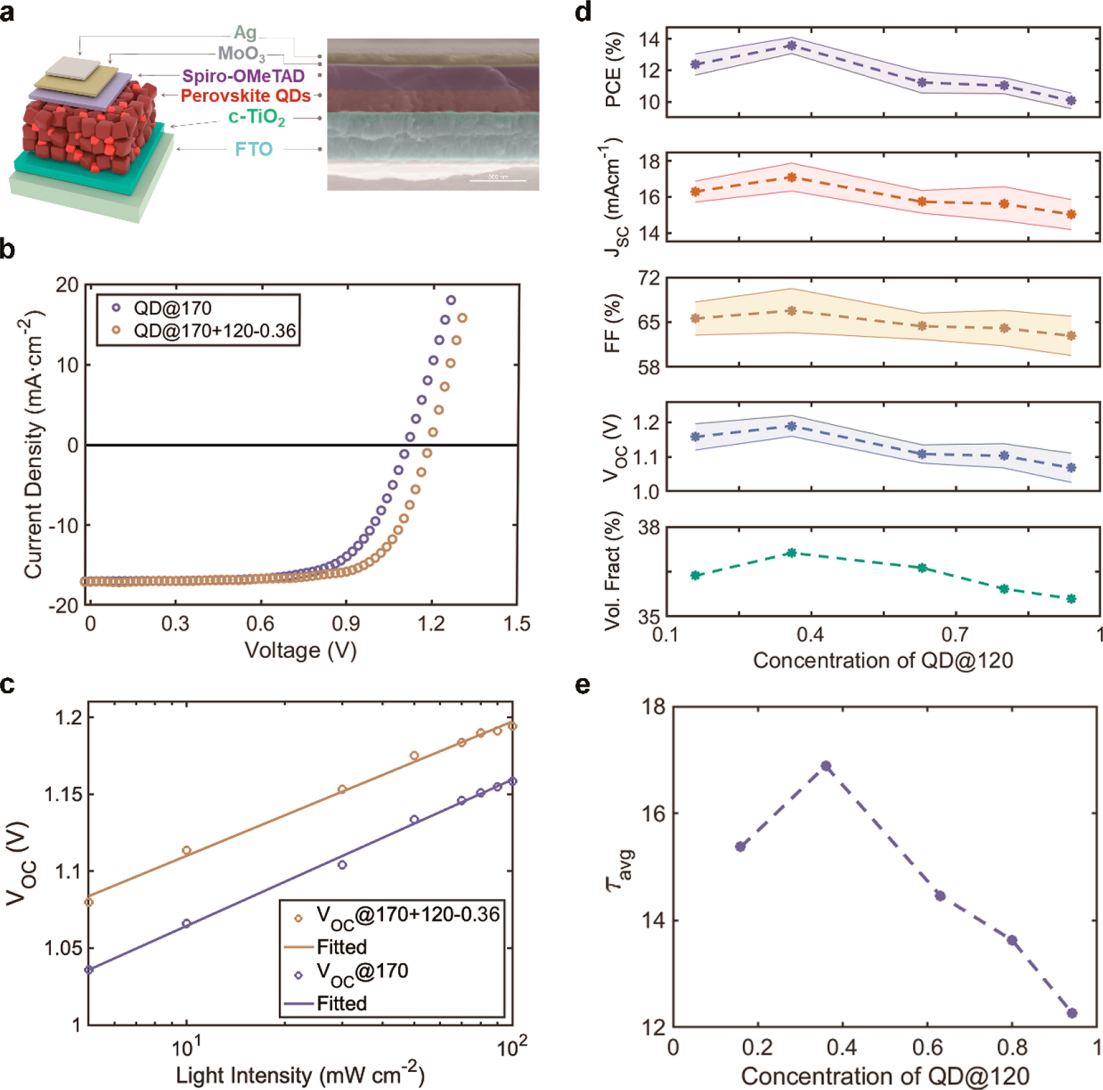
PV performance of a PQD device and charge carrier property. (a) Schematics and SEM cross section image of a PQD solar device. (b) *J*–*V* curves and (c) light-intensity dependent *V*_OC_ of best-performing PQD devices made with QD@120-0 and binary-sized QD films@170+120-0.36. (d) Photovoltaic parameter evolution with different concentrations of QD@120. Statistics from 50 individual devices with error bar shown as the corresponding shaded area. (e) Corresponding average carrier lifetime.

To investigate the influence of binary-disperse packing on device performance, we fabricated PQD solar cells with a planar structure of FTO/c-TiO_2_/PQD/Spiro-OMeTAD/MoOx/Ag, as illustrated in Figure 4a. Fabrication details can be found in the Methods. To facilitate the charge transport within the QD film, the long insulating organic ligands OAm^+^ and OA^–^ were replaced by shorter ligands, FA^+^ and OAc^–^,^24^ following previous literature.^38^Figure S11 and Table S4 present GISAXS results and fitted volume fractions of the blend films after the ligand exchange process. The overall volume fractions slightly increase for all different number ratios, as a result of the reduction of ligand lengths, while the trend of volume fraction variation versus the concentration of QD@120 remains the same, in line with the device performance (Table S5). The device fabricated with the binary-size film with the highest volume fraction (concentration of QD@120 = 0.36) delivered the superior efficiency of 14.42%, *J*_SC_ of 17.08 mA cm^–2^, *V*_OC_ of 1.19 V, and FF of 71.12% (Figure 4b and Table S5). In comparison, the PV device fabricated with pure QD@170 presented a *J*_SC_ of 17.02 mA cm^–2^, *V*_OC_ of 1.11 V, FF of 67.45%, and PCE of 12.75% (Figure 4b and Table S5). The device performance improvement is mainly reflected in the *V*_OC_ and FF (Figure 4d).

UV–vis absorption (Figure S12) and ultraviolet photoelectron spectroscopy (UPS) (Figure S13) measurements were performed to determine the optical bandgap and the band structure of the films (Figure S14). The absorption edge of QD@170 (1.73 eV) shows a red shift from that of QD@120 (1.81 eV), consistent with PL results, while the binary-size film shows an absorption edge the same as that of the film of QD@170. Hence, the quantum confinement effect leads to a slightly larger bandgap of QD@120 compared to QD@170 (Figure S14). This could potentially impede electron transport from QD@170 to QD@120 while facilitating electron transport in the reverse direction. Therefore, the presence of adequate carrier transport pathways may enable the mixture of two differently sized PQDs to effectively mitigate recombination, being beneficial to voltage enhancement. This is analogous to the bulk heterojunction structure employed in organic solar cells, where a combination of two or three organic donor and acceptor materials could promote charge transport, suppress recombination and, consequently, reduce voltage loss,^39−41^ despite uneven energy level distribution. To further confirm this, we measured time-resolved photoluminescence (TRPL) to determine the carrier lifetime. The TRPL curves (Figure S15) were fitted with a biexponential function, and the average carrier lifetime (Table S6) is plotted in Figure 4e. The device with the highest volume fraction also exhibited the longest average carrier lifetime, leading to a reduction of trap-assisted recombination and, therefore, contributing to reduced *V*_OC_ loss and FF enhancement. Last, the recombination mechanism of the binary-disperse films was further studied by the light-intensity-dependent open-circuit voltage (Figure 4c). The curves were fitted with the relation  in the linear-log scale, where *n* is the ideality factor of a diode, *k* is the Boltzmann constant, *T* is the absolute temperature, *q* is the elementary charge, and *C* is a constant.^42,43^ Thus, the ideality factor can be determined from the slope of the curve. The ideality factor of the best device, QD@170+120-0.36, is 1.48, smaller than that of the pure QD@170 device (1.61), suggesting a much lower trap-assisted recombination, in agreement with TRPL results. Moreover, we performed the space-charge-limited current (SCLC) test to investigate the trap density difference (Figure S16).^27^ The trap density was calculated by , where the ε_r_ is the relative dielectric constant of CsPbI_3_, ε_0_ is the vacuum permittivity, *V*_TFL_ is the trap-fill limited voltage, *e* is the elementary charge, and *L* is the thickness of the film. The calculated trap density of the binary film is 3.00 × 10^15^ cm^–3^, smaller than that of the pure QD@170 film, confirming that the trap density is lowered with higher packing density. The electron mobility was also calculated by the Mott–Gurney law,^44^, where μ is the mobility. Mobility values of 8.78 × 10^–4^ cm^2^ V^–1^ s^–1^ and 7.61 × 10^–4^ cm^2^ V^–1^ s^–1^ were obtained for binary and control films, respectively, demonstrating the improvement in charge transport. Electrochemical impedance spectroscopy (EIS) measurements were carried out to further elucidate the recombination mechanisms. Figure S17 presents the EIS curves of control and binary devices measured under illumination at a bias of 1.1 V. The curves were fitted to the equivalent circuit as shown in Figure S18a, and the transport and recombination resistance were gained from the fitting results and plotted in Figure S18b.^45^ Comparing to the control device, the transport resistance of the binary film device is smaller while the recombination resistance is larger, further confirming that denser packing promotes charge transport and suppresses recombination.

## Conclusions

In summary, this study highlights the significance of packing density in PQD-based solar cells and provides a facile method, binary-disperse mixing, which effectively increases the packing density of perovskite quantum dots, and thus obtained largely suppressed trap-assisted recombination and much longer carrier lifetime. As a result, the binary-disperse PQD solar device with QD@170+120-0.36 achieved a superior PCE of 14.42% as a result of *V*_OC_ and FF enhancements. The packing density of the binary-component region showed a bowing trend, which is lower at two ends while it maximizes in the middle. Apart from these, MD simulations suggest that organic ligands prefer to attach to the edges and corners of a PQD. Therefore, smaller-sized PQDs with a larger percentage of atoms residing on the edge/corner have higher ligand densities than larger-sized PQDs. The enhanced adaptability provided by these ligands may help the former PQDs better meet demanding packing requirements and assist them in establishing more face-to-face contact. Binary-disperse mixing could further promote the face-to-face contact frequency, thereby giving rise to a higher packing density.

## Methods

### Materials

Oleic acid (OA, analytical reagent), oleylamine (OAm, technical grade 80–90%), and 1-octadecene (ODE, technical grade 90%) were purchased from Aladdin Bio-Chem Technology Corp. Cesium acetate (CsAc, 99.99%) and τitanium(IV) isopropoxide (TIPP, 99.999% trace metals bases), octane (anhydrous, ≥99%), methyl acetate (MeOAc, anhydrous 99.5%), 4-*tert*-butylpyridine (tBP, 96%), chlorobenzene (anhydrous, 99.8%), acetonitrile (anhydrous, 99.8%), molybdenum(VI) oxide (MoOx, 99.97% trace metals basis), bis(trifluoromethane) sulfonimide lithium salt (Li-TFSI, 99.95% trace metals basis), and FK 102 Co(III) TFSI Salt (98%) were purchased from Sigma-Aldrich. Lead(II) iodide (PbI_2_, 99.99%) was purchased from Xi’an Polymer Light Technology Corp. Hexane (GR grade 95%) was purchased from DUKSAN. Spiro-OMeTAD was purchased from YingKou YouXuan Technology Corp. HCl (37%) HNO_3_ (68%), and EtOH were purchased from RCL Labscan Limited. All of the chemicals were used without further purification.

### Synthesis and Purification of CsPbI_3_ QDs

The CsPbI_3_ quantum dots were synthesized via a modified hot injection method using Schlenk technology.^27^ Particularly, 5 mmol of CsAc powder was dissolved in 10 mL of oleic acid (OA) at 80 °C under inert atmosphere and kept at 50 °C before use. One millimole of PbI_2_ powder was dissolved in 10 mL of 1-octadecene (ODE) in a three-neck flask. The mixture was heated to 120 °C before 1.25 mL of oleic acid (OA) and 1.25 mL of oleylamine (OAm) were added. The solution was held at the temperature and degassed for 1 h to remove the moisture. Thereafter, the precursor was adjusted to the desired temperature (in this case, the synthesis temperatures were chosen to be 170 and 120 °C). Once the temperature was reached, 0.4 mL of Cs-OA solution was swiftly injected to the flask, and the reaction was continued for 5–10 s. The whole reaction was quenched immediately by immersion in an ice bath. The QD solution should be red in color with its luminance turning from dark to light as the synthesis temperature drops. The QDs were purified by adding 30 mL of MeOAc (ratio of QD solution/MeOAc = 3:7) and then centrifuged at 7500 rpm for 3 min. The precipitated QDs were redispersed in hexane and washed again with MeOAc (the ratio of QD solution/hexane/MeOAc = 1:1:1) and centrifuged at 7500 rpm for 3 min. The precipitates were dissolved in hexane and stored overnight at −4 °C. For QDs synthesized at 120 °C, the supernatant from the last washing cycle was kept while the precipitates were discarded. An additional washing process was applied to the supernatant with the ratio of QD solution/hexane/MeOAc = 1:1:1.5. The purpose of this step is to filter out the QDs with the desired size. All QD solutions were centrifuged at 3000 rpm for 3 min to remove excess impurities (e.g., Pb-oleate etc.). The supernatants were dried and dissolved in octane at a concentration of ∼70 mg/mL.

### Device Fabrication

The structure of the solar device in this study was chosen to be FTO/c-TiO_2_/PQDs/Spiro-MeTAD/MoOx/Ag. Specifically, the compact TiO_2_ layer was deposited via spin-coating TIPP solution (0.9 mL TIPP and 100 μL HCl (37%) dissolved in 15 mL EtOH and stirred in a glovebox overnight) on a prepatterned FTO substrate at 4000 rpm for 30 s. Then, the substrate was annealed by sintering in a Muffle furnace at 500 °C for 1 h and cooled down before use. The deposition of the perovskite layer was performed in a drybox with relative humidity between 15% and 20%. Before coating with the PQD layer, a thin layer of saturated CsAc/MeOAc solution was deposited onto the substrate for the sake of passivation and better charge transport. Subsequently, the prepared QD solution was spin-coated onto the c-TiO_2_/FTO substrate at 1000 rpm for 10 s, followed by spin-coating at 2000 rpm for 10 s. The binary-sized QD film was fabricated via mixing the two kinds of QDs at a certain number ratio. The anion ligand exchange then proceeded in a drybox by dropping 100 μL of MeOAc onto the film and immersing for 7 s before spin-coating. The spin-coating and anion ligand exchange processes were repeated 4 times to obtain a PQD film with enough thickness. After that, the cation ligand exchange was processed in the glovebox by dropping 100 μL of FAI/EtOAc saturated solution onto the film and immersing for 10 s before spin-coating. After the ligand exchange, the film was placed on the hot plate and heated to 50 °C to remove excess solvent. The Spiro-oMeTAD (72.3 mg in 1 mL of chlorobenzene with 28.8 μL of tBP, 17.5 μL of Li-salt, and 24 μL of Co-salt; the Li-salt was prepared as 520 mg of Li-TFSI in 1 mL of acetonitrile; the Co-salt was prepared by dissolving 300 mg of FK 102 Co(III) TFSI Salt in 1 mL of acetonitrile) was then spin-coated on the device with a speed of 5000 rpm for 30 s. Finally, a thin layer of MoOx (around 5 to 6 nm) as a buffer layer was vacuum deposited before the Ag electrode was deposited.

The fabricating conditions of the PQD film such as spin coating speed and time, the immersion time for ligand exchange, and the layer of PQDs were first optimized in the fabrication of mono-PQD films and then applied to binary-PQD films. The deposition process for monofilm and binary film are designed to be the same to minimize the effects from the film deposition.

### Characterization

TEM was measured by FEI Tecnai G2 operating at 120 kV. The synthesized PQDs were dissolved in hexane to form a solution whose concentration is 1 mg/mL and dropped on a carbon-film-supported Cu grid. The SEM cross-section image of the fabricated solar device was measured by a FEI Quanta 480F with an operating voltage of 10 kV. The solution PL was measured by a SpectraMax M5. The concentration of PQD solution was 1 mg/mL in hexane. The steady-state film PL spectra were measured at room temperature using a commercially available Raman/PL spectrometer (Horiba, Inc.) with a 532 nm laser source. Samples were fabricated on glass substrates. The time-resolved photoluminescence (TRPL) measurements were conducted with an FLS920P Edinburgh Analytical Instrument equipped with a 485 nm diode laser (EPL-485) as the excitation source. GIWAXS measurements were carried out with a Xeuss 2.0 SAXS/WAXS laboratory beamline using a Cu X-ray source (8.05 keV, 1.54 Å) and a Pilatus3R 300 K detector. GISAXS measurements were conducted at the TLS 23A small- and wide-angle X-ray scattering (SWAXS) beamline (10 keV, 1.24 Å) at the National Synchrotron Radiation Research Center, Taiwan. The film samples were deposited on a Si substrate, and the incidence angle was 0.3°. The current density–voltage (*J*–*V*) curves were measured by a Keithley 2612 source meter unit under an AM 1.5G solar simulator (100 mW cm^–2^). The UV–vis absorption was taken on a Hitachi U-3501 ultraviolet/visible/near-infrared spectrophotometer. The PQD films were deposited on a glass substrate and encapsulated before measuring.

## Supporting Information Available

Theoretical modeling, MD simulation, and additional figures (PDF)
