# Supplementary material for: Fostering the Dense Packing of Halide Perovskite Quantum Dots through Binary-Disperse Mixing
Source: ACS Nano. 2023 Oct 3;17(20):20634–42. doi: 10.1021/acsnano.3c07688 (PMC10604077; doi:10.1021/acsnano.3c07688)
Supplement: Supplementary file 1 — nn3c07688_si_001.pdf [file nn3c07688_si_001.pdf]

Supplementary Information

Fostering the Dense Packing of Halide Perovskite Quantum Dots through Binary-Disperse Mixing

Shiang Li[1], Ziqi Wang[1], Yuhao Li[1,2], Chun-Jen Su[3], Yuang Fu[1], Yi Wang[1,], Xinhui Lu[1,*]*

1Department of Physics, The Chinese University of Hong Kong, 999077 Hong Kong SAR, China

2Spallation Neutron Source Science Center, Institute of High Energy Physics, Chinese Academy of Sciences, Dongguan 523803, China

3National Synchrotron Radiation Research Center, Hsinchu Science Park, Hsinchu 30076, Taiwan

*Email: xinhui.lu@cuhk.edu.hk (X. L.)

*Email: yiwang@cuhk.edu.hk (Y. W.)

Theoretical modelling: The gracing-incidence SAXS data processing step contains model building and data fitting process.[1, 2] The scattering intensity can be quantified by the following formula:

$$I(q) = A\langle P(q, \overline{R})\rangle S(q) \qquad \text{(S1)}$$

The first product $A\langle P(q, \overline{R})\rangle$ is the Rayleigh function, with A a constant proportional to particle volume fraction $\eta = \pi\rho d^3/6$, where ρ and d are the number density and diameter, respectively; and $\langle P(q, \overline{R})\rangle$ is the average form factor which characterizes the intra-particle property of a system. One simplification adopted is that the shape of the PQDs is treated as spheres,[2, 3] therefore $\langle P(q, \overline{R})\rangle$ is given by:

$$\langle P(q, \overline{R})\rangle = \int_0^\infty P(q, R) D(R, \overline{R}) dR \qquad \text{(S2)}$$

where $P(q, R)$ is the form factor of spherical particles and described by a spherical Bessel function:

$$P(q, R) = j_1(qR) \qquad \text{(S3)}$$

$$j_1(x) = \left[\frac{3(\sin x - x\cos x)}{x^3}\right]^2 \qquad \text{(S4)}$$

And $D(R,\bar{R})dR$ is to describe the probability of particles with a mean radius $\bar{R}$ having the radius between $[R,\ R + dR]$, where $D(R,\bar{R})$ is the Schultz distribution[2, 4, 5]:

$$D(R,\bar{R}) = \left(\frac{Z+1}{\bar{R}}\right)^{Z+1} \frac{R^Z e^{-(Z+1)R/\bar{R}}}{\Gamma(Z+1)} \qquad \text{(S5)}$$

with $Z > -1$.

$S(q)$ is the structure factor which describes statistically the contribution of inter-particle correlation to $I(q)$. In this study, a hard sphere structure factor with Percus-Yevick (P-Y) closure was implemented, for it provides a reasonable approximation of uncharged particles that freely rotate and have a moderate polydispersity. To gain an insight of the derivation of structure factor, it is reasonable to evoke the Ornstein-Zernike (O-Z) equation.[6, 7] Fourier transforming the O-Z equation leads to the following:

$$h(q) = c(q) + \rho c(q)h(q) \qquad \text{(S6)}$$

where $h(q)$, $c(q)$ are the total correlation function and direct correlation function, respectively, in q space. The structure factor therefore becomes:

$$S(q) \equiv 1 + \rho h(q) = \frac{1}{1 - \rho c(q)} \quad \text{(S7)}$$

Defining $g(r)$ as the radial distribution function gives the P-Y approximation the form of:

$$g(r)\left(e^{-\beta U(r)} - 1\right) = e^{-\beta U(r)} c(r) \quad \text{(S8)}$$

with $U(r)$ the pair potential which takes the following form for hard spheres

$$U(r) = \begin{cases} \infty, & r \leq 2R \\ 0, & r > 2R \end{cases} \quad \text{(S9)}$$

In a mono-disperse system, the Fourier transform of $c(r)$ combined with (S7) leads to an analytical expression of $S(q, R)$

$$S(q, R) = \frac{1}{X^2(q, R) + Y^2(q, R)} \quad \text{(S10)}$$

with

$$X(q, R) = 1 - 12\eta\left[Af_1(q, R) + Bf_2(q,R)\right] \quad \text{(S11)}$$

$$Y(q, R) = -12\eta\left[Af_3(q, R) + Bf_4(q,R)\right] \quad \text{(S12)}$$

where A and B are coefficients related to volume fraction η, and f_i is a function of reduced wave number $y = 2qR$.[7]

The single component film with particle radius R_1 and number density ρ_1 can be well described by the above structure factor. However, if a second component with radius R_2 and number density ρ_2, based on which $\alpha \equiv R_1/R_2$ ($\alpha \in [0, 1]$) and $x \equiv \rho_2/(\rho_1 + \rho_2)$ could be defined, was added to form a binary disperse system, the interaction between particles should contain three parts as well as the correlation function, which were solved from P-Y equation by J. L. Lebowitz[8] and N. W. Ashcroft and his co-workers[9]:

$$-c_{11}(r) = a_1 + b_1 r + dr^3, \;\; r < 2R_1$$

$$-c_{22}(r) = a_2 + b_2 r + dr^3, \;\; r < 2R_2$$

$$-c_{12}(r) = a_1, \;\; r < \lambda$$

$$= a_1 + [b\Lambda^2 + 4d\lambda\Lambda^3 + d\Lambda^4]/r, \;\; \lambda < r < (R_2 + R_1)$$

(S13)

with $\Lambda \equiv r - \lambda$ and $\lambda \equiv R_2 - R_1$; and a_i, b_i, b and d are all functions of η, α, and x (details can be found in Appendix). The Fourier transform proceeds as:

$$c_{ij} = \frac{4\pi}{q}\int c_{ij}(r)\sin(qr)dr \qquad \text{(S14)}$$

And the general form of structure factor can be constructed:

$$S_{ij} = (N_i N_j)^{-1/2} \left\langle \sum_{n,m} e^{i\boldsymbol{q} \cdot (\boldsymbol{r}n^i - \boldsymbol{r}m^j)} \right\rangle - (N_i N_j)^{1/2} \delta_{q,0} \qquad \text{(S15)}$$

Derived from the relation of $c_{ij}(q)$ and $S_{ij}(q)$, the explicit solutions can be described as:

$$S_{11}(q,R) = \left\{1 - \rho_1 c_{11}(q, R) - \rho_1 \rho_2 c_{12}^2(q,R)/[1 - \rho_2 c_{22}(q,R)]\right\}^{-1}$$

$$S_{22}(q,R) = \left\{1 - \rho_2 c_{22}(q, R) - \rho_1 \rho_2 c_{12}^2(q,R)/[1 - \rho_1 c_{11}(q,R)]\right\}^{-1}$$

$$S_{12}(q,R) = (\rho_1 \rho_2)^{1/2} c_{12}(q,R) \left\{[1 - \rho_1 c_{11}(q, R)][1 - \rho_2 c_{22}(q,R)] - \rho_1 \rho_2 c_{12}^2(q,R)\right\}^{-1}$$

(S16)

The above structure factor is used here to model the bi-disperse system.

GISAXS fitting: The GISAXS data of PQD film with single component were fitted using a spherical form factor and a mono-dispersed hard sphere structure factor. From the Theoretical Modelling section, one can acquire the fitting parameter of mono-dispersed system:

$$I(q,R,Z,\eta) \propto aP(q,R,Z)S(q,R,\eta) \qquad \text{(S17)}$$

with a as the pre-factor related to particle volume and number density. The GISAXS data of binary

PQD components were fitted using a spherical form factor and a bi-dispersed hard sphere structure factor. The fitting parameters of the theoretical model:

$$I(q,R,Z,\eta,\alpha,c) \propto \sum_{i,j} a_{ij} P_{ij}(q,R,Z) S_{ij}(q,R,\eta,\alpha,c) \qquad \text{(S18)}$$

where

$$P_{ij}(q,R,Z) = \sqrt{P_{ii}(q,R,Z) \times P_{jj}(q,R,Z)} \qquad \text{(S19)}$$

For the film GISAXS measurement the incident angle is 0.3˚.

MD Simulations: All-atom (AA) molecular dynamics (MD) simulations were first employed to investigate ligand conformation and distribution over the PQD surface in octane. Same as in the experiments, OAm+/OA- were modeled as the long-chain ligands and FA^+/OAc^- as the short-chain ligands. Four cubic PQD models were constructed, which consisted of 4.5, 6.5, 8.5 and 10.5 unit cells, respectively, where each cubic unit cell had a dimension of 0.62 nm. Given the stability of lead halide octahedral within various perovskite-related materials,[10, 11] CsI-rich (100) crystallographic planes were chosen to represent the PQD surface, consistent with previous modeling studies. The surfaces of PQDs were found to be passivated by ammonium in previously proposed models, which explained their high tolerance towards degradation. Following these

studies, in our PQD model,[12, 13] 50% of surface Cs^+ atoms were replaced by pre-inserted OAm^+. A given PQD was then placed in a simulation box with at least 4 nm between its surface and the box boundaries. The anionic ligands OA^- and the solvent octane molecules were randomly inserted into the box. To mimic experimental conditions, the molar ratio of OA^-:octane was set to 1:16. Free cationic OAm^+ ligands were then inserted into the system to ensure that the final simulation system (**Figure S3**) was charge-neutral. The simulation systems of PQDs with short-chain ligands were constructed using the equilibrated PQD-long-ligands structures as templates. The long-chain ligands attached to the PQD surface were replaced by the short-chain ligands, mimicking the ligand exchange effect in the corresponding experiments.

The CHARMM36[14] and CHARMM General Force Field (CGenFF)[15, 16] were adopted in all AA simulations. The CHARMM-GUI[17] and CGenFF program[18, 19] were used to model ligands without existing parameters. For the FA^+ molecule that received a high penalty score from the CGenFF program, additional optimization was carried out using the VMD Force Field Toolkit Plugin (FFTK)[20] and Gaussian[21]. Nonbonded parameters for Cs^+ and I^- ions were adopted from Joung et al.[22] and Pb^{2+} parameter was adopted from Li et al.[23] After energy minimization all systems were equilibrated for 1 ns in the NVT ensemble, followed by 20 ns equilibration in the NPT ensemble.

Simulated annealing was then performed in which the systems were heated up from 300 to 500K at the rate of 50 K/ns; then the temperature was kept at 500K for 1 ns and reduced to 300 K at the same rate of 50 K/ns. After another 20 ns equilibration, the resulting structures were simulated for 100 ns in the NPT ensemble as the production run. For each PQD model, four replica production runs were performed.

In all AA simulations, van der Waals interactions were smoothly switched off from 0.8 nm to 0.9 nm, while electrostatic interactions were calculated using the particle mesh Ewald (PME) method with a cutoff of 0.9 nm. The temperature of all systems was maintained at 300 K by velocity rescaling with a stochastic term[24] and Berendsen coupling was used to maintain the pressure at 1 bar. All bonds with hydrogen atoms were constrained using the LINCS algorithm. All simulations were performed with the GROMACS software package[25] and visualized using the Visual molecular dynamics (VMD) program[26]. The 3D electrostatic potentials shown in **Figure S5** were evaluated using the VMD PMEPot Plugin[27].

To construct a model mimicking a PQD after spin coating, when most of the solvent and free ligands were cast off, only ligands within 2 nm of a PQD were retained from the aforementioned simulations. More specifically, two such PQD models each with a size of 6.5-unit cells were

retained, neutralized and then simulated for 100 ns in the NVT ensemble, during which they were only allowed to move in the surface-to-surface orientation shown in **Figure 3c**. Finally, the surface ligand density on a PQD was defined as the number of ligand heads divided by the PQD surface area. Measurement of the normalized mass density of ligands was performed by first aligning the PQD to the center of the simulation box. The ligand densities were then scanned and averaged in the ±x, ±y, and ±z directions over the duration of the four replicas of production runs.

To further investigate the interactions between multiple PQDs, we next conducted coarse-grained (CG) simulations using the MARTINI3 force field. Large PQDs (10.5-unit cell) and small PQDs (6.5-unit cell) with long-chain ligands were constructed based on their equilibrated AA models without the octane solvent or ligands beyond 2 nm of a PQD surface. Altogether three CG systems were constructed: all-large (40 large PQDs), all-small (70 small PQDs), and 1:1-mix (25 large and 25 small PQDs). The CG models of these PQDs were inserted in a vacuum box of 50*50*60 nm^3 and neutralized by additional long-chain ligands. A baseboard (carbon atoms, 10 atoms/nm^2) was placed in the x,y-plane at z = 0, while a flat bottom potential was placed for z > 55 nm to prevent the particles from crossing the top periodic boundary. CG parameters of OAm^+ and OA^- were obtained from the DOPC molecule in MARTINI3 while nonbonded parameters of PQDs were

retained from their all-atom models. All systems were equilibrated for 100 ps in an NVT ensemble after energy minimization and then simulated in the NVT ensemble for 40ns as the production run. Each system at a given large:small PQD number ratio (1:0, 1:1, and 0:1 for the all-large, 1:1-mix and all-small systems, respectively) was simulated for N = 40 replicas, resulting in a total CG simulation time of ~5 μs.

Based on the CG simulations we measured the probability distribution function $P(r)$ of the minimum distance dm between any two PQDs (excluding ligands):

$$P(r) = \frac{\sum_{i=1}^{N} N_{r=d_m,i}}{\sum_{i=1}^{N} N_{pair,i}} \quad \text{(S20)}$$

where $N_{r=d_m,i}$ is the number of PQD pairs with their minimum distance in the range $[r, r+0.2nm)$ from the i-th simulation replica, and $N_{pair,i}$ is the total number of PQD pairs from the i-th simulation replica. Next, for each Cs atom (denoted as atom A) on the surface of a given PQD, we measured the frequency with which it came into contact ($d_m \leq$ 4 nm, see **Figure S9**) with a Cs atom (denoted as atom B) of a second PQD. We further distinguished the location of atom B based on whether it was on the edge, corner, face (anywhere on a PQD surface that is not the edge or corner), or any of the above three locations of a PQD. The resulting contact maps are thus labelled as

‘edge’, ‘corner’, ‘face’ or ‘any’, respectively. For a given atom A at (a,b,c), its contact frequency with atom B of a second PQD is therefore:

$$P(a,b,c) = \frac{\sum_{i=1}^{N} \sum_{j=1}^{n_{st}} K_{i,j}}{N n_{st}} \qquad \text{(S21)}$$

$$K_{i,j} = \begin{cases} 0, & d_m(a, b,c) > 4\ nm \\ 1, & d_m(a, b,c) \leq 4\ nm \end{cases} \qquad \text{(S22)}$$

where n_{st} is the number of PQDs with a given size type (st) (large/small), N = 40 is the number of simulation replicas and d_m is the minimum distance between atom A at (a,b,c) and atom B (edge/corner/face/any) that belongs to a second PQD. More specifically, we set the center of the first PQD to the origin (0,0,0) and place its edges parallel to the x, y, and z axes. Denoting the edge length of the PQD as 2L, we obtain the coordinates of atoms on its surface as $(\pm L, x,y)$, $(x, \pm L,y)$ or $(x,y, \pm L)$, where $-L \leq x,y \leq L$. Due to symmetry, the computed contact frequency at (a,b,c) can be mapped onto $P_{0 \leq x \leq y \leq L}(x,y,L)$ where $(x,y,L) = sorted(|a|,|b|,|c|)$. The averaged $P_{0 \leq x \leq y \leq L}(x,y,L)$ is then mapped onto a $[-1, 1]$ by $[-1, 1]$ square with $-1 \leq x,y \leq 1$, while missing data at Cs atoms substituted by ligands was obtained via 2D interpolation. Finally, the results are smoothed by 2D interpolation and normalized to yield the contact maps shown in **Figure S10.**

Finally, with $K_{i,j}$ defined in the previous equation, the contact ratio of a given PQD surface was obtained as: $R_{i,j} = \frac{\sum_{x=0}^{L}\sum_{y=0}^{L} K_{i,j}(x,y)}{\mathrm{L}^2}$, where missing data due to ligand substitution were obtained via linear interpolation. For each PQD, the maximum contact ratio among its six surfaces was then computed and shown in **Figure 3f**.

Ligand density from NMR and ICP-OES measurement: The ligand density is estimated as the number of ligands attached per area on a PQD surface:

$$\rho_{ligand} = \frac{c_{ligand}}{c_{PQD} \times a^2 \times 6} \qquad \text{(S23)}$$

where c_{ligand} and c_{PQD} are the molar concentration of ligands and PQDs, respectively; and a is the average edge length of PQDs.

The molar concentration of ligands was determined by NMR measurement. Specifically, a certain amount of dried QD with a mass of m_1 was dispersed in v_1 volume of deuterated chloroform. By setting pure oleyl species as the standard sample in NMR test, the c_{ligand} was acquired via the digital ERETIC[28].

The c_{PQD} was measured and calculated via the ICP-OES technique.[29] The same amount of dried

QDs was dissolved in thick nitric acid (69% in vv) and diluted to 1%. The prepared solution was then to perform the ICP-OES test which give the concentration of Pb^{2+} ion in the solution. Under the assumption that all the Pb^{2+} were sourced from the PQDs, the quantity of $CsPbI_3$ c_{CsPbI3} was thus gained. With the lattice constant of PQDs d given, the amount of QDs can therefore be obtained:

$$c_{PQD} = \frac{c_{CsPbI3} \times d^3}{a^3} \tag{S24}$$

Combining c_{ligand}, c_{CsPbI3}, and formula S23 and S24, the ligand density can be calculated.

Softness: For a core-shell structure particle, its softness is given by the ratio of the length of the ligand and the diameter or edge length of the particle.[30] Specifically, if a particle with a core diameter of d and covered with a ligand whose length is l, the softness for the particle is

$$\lambda \equiv \frac{2l}{d} \tag{S25}$$

To analyze ligand conformation and distribution, the normalized mass densities of OAm^+ and OA^- were measured from all-atom MD simulations as a function of their distance to a PQD surface. As shown in **Figure S19**, the normalized mass densities reach the bulk values at 1.9, 1.9, 1.9, 2.0 nm

for PQDs of 4.5, 6.5, 8.5, and 10.5 unit cells, respectively. Therefore, the thickness of the long-chain ligand layer was taken to be approximately 2 nm regardless of the PQD size. Based on further simulations mimicking the experimental conditions after spin coating, the average distance between two PQDs with attached long-chain ligands was found to be 2.1 nm (**Figure 3c**), reflecting ligand interpenetration between neighboring PQDs. Based on this average surface-to-surface distance between two PQDs, an average ligand length of 1.05 nm was estimated for the PQD film. The softness values for QD@170 and QD@120 were therefore calculated to be 0.167 and 0.242, respectively. The total volume fractions of the core-shell PQDs thus reached 55.1% and 68.4%, respectively. It is worth mentioning that, after considering ligands in the calculation of volume fraction, the packing densities of the pure QD@170 and pure QD@120 are comparable to the limit of random packing[31].

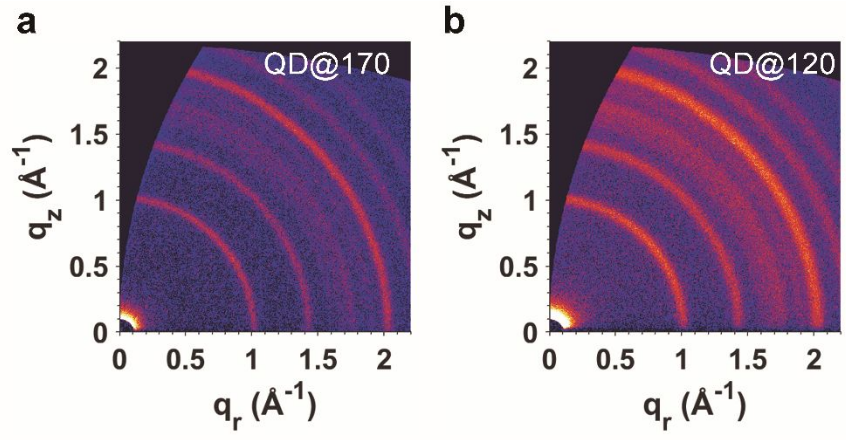

Figure S1 GIWAXS 2D patterns of QD films made of (a) pure QD@170 and (b) pure QD@120, respectively.

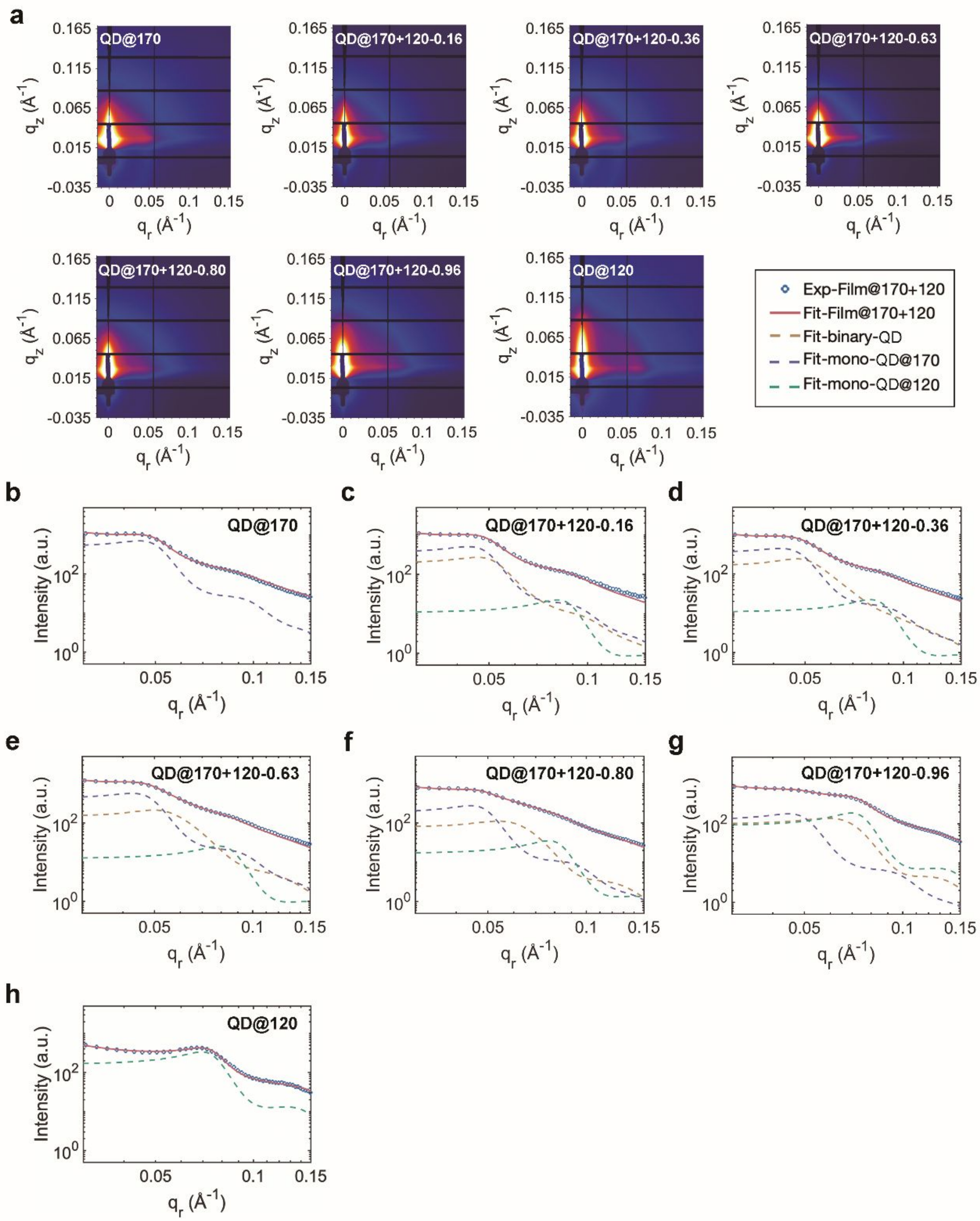

Figure S2 GISAXS 2D patterns and linecut curves. (a) GISAXS 2D patterns of pure QD films and blend film with various mix number ratios. (b) to (h) are the linecut fitting of pure QD films and blend films with various number ratios. The samples are labelled with the film components and distinguished by the suffixes that correspond to the concentration of QD@120 in the film.

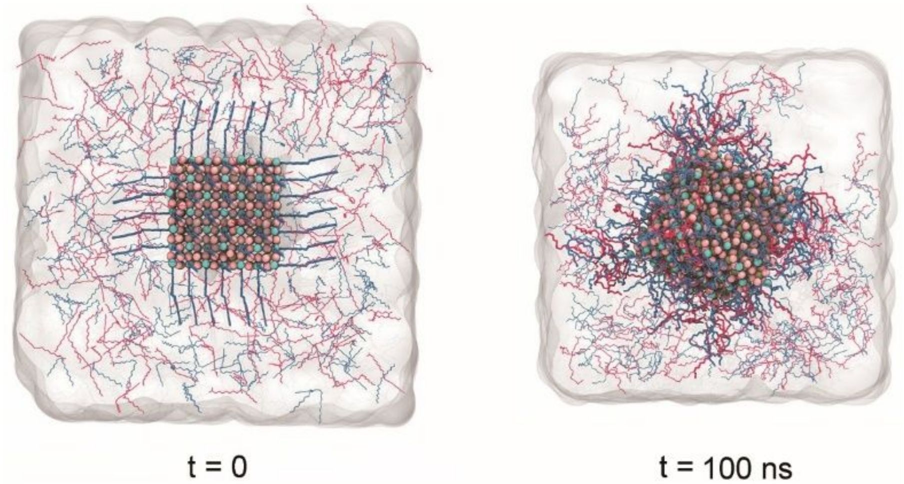

Figure S3 Simulation snapshot of representative initial and final structures of a single PQD in octane. The PQD (edge length = 6.5-unit cell) is attached with long-chain ligands. The PQD is represented by beads: Cs^+ (cyan), Pb^{2+} (yellow) and I^- (pink), while OA^- (red) and OAm^+ (blue) are shown in thin lines, with the pre-inserted OAm^+ and any ligand whose head group is within 1 nm of the PQD at t=100 ns represented by thick sticks. The octane solvent is represented by transparent surfaces.

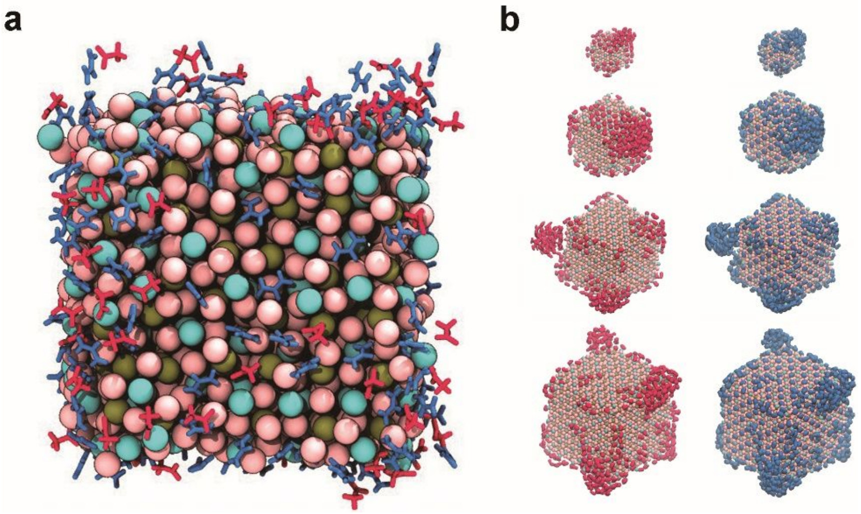

Figure S4 (a) Simulation snapshot of a single PQD with short-chain ligands in octane. OAc- and

FA+ are colored in red and blue, respectively, while octane is omitted for clarity. (b) 3D occupancy iso-surfaces (5%) of the OAc^{-} (red) and FA^{+} molecules (blue) of a given PQD in all-atom MD simulations with octane. From top to bottom, the size of the PQD is 4.5, 6.5, 8.5, and 10.5 unit cells, respectively.

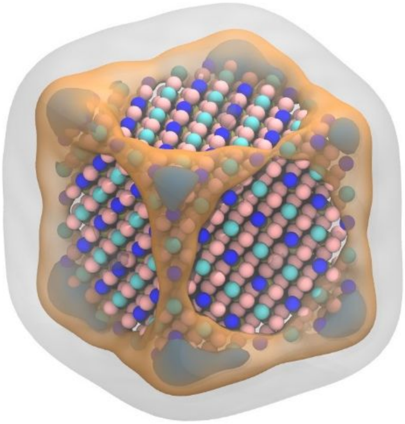

Figure S5 3D electrostatic potentials of a single 6.5-unit sized PQD with pre-inserted OAM^{+} computed using the PMEpot plugin[26] of VMD [1.9.4]. The sliver, orange, and blue transparent surfaces represent increasingly strong (positive) electrostatic potential. The PQD is represented by beads (Cs^{+}: cyan, Pb^{2+}: yellow and I^{-}: pink) and for clarity, only the nitrogen atoms of pre-inserted OAm^{+} are shown (dark blue).

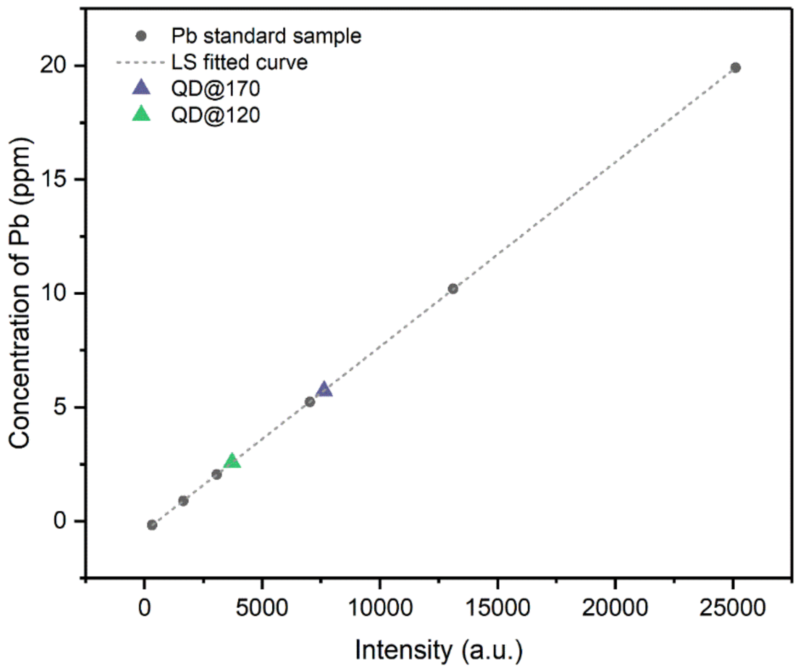

Figure S6. Interpolation method to determine the concentration of Pb in QD solutions via ICP-OES.

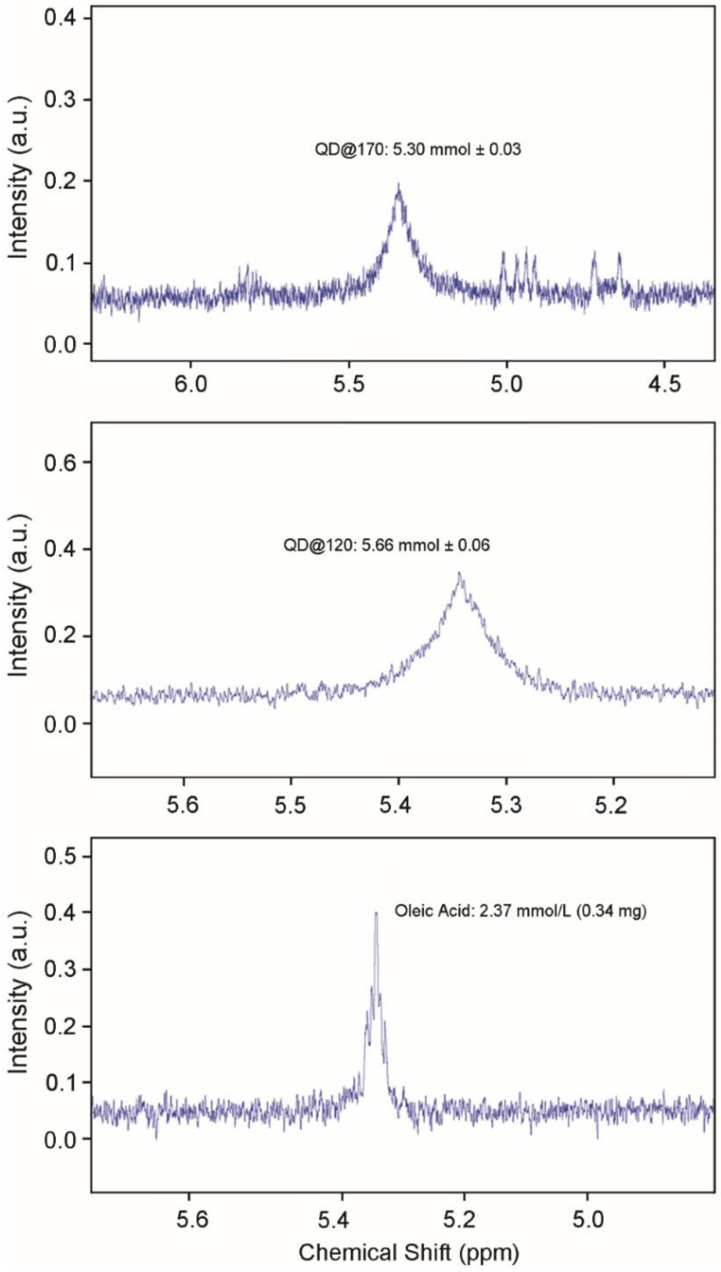

Figure S7. The concentration of attached ligands determined by ERETIC technique via NMR spectra.

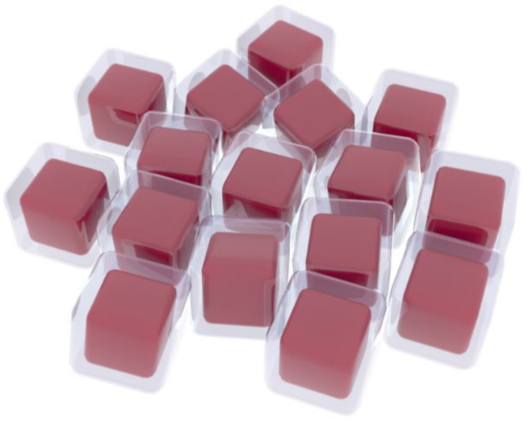

Figure S8. Illustration of softness. The red cube cores represent the QDs while the transparent shells represent the surface ligand as they are assumed to be “transparent” under X-ray due to the low electron density.

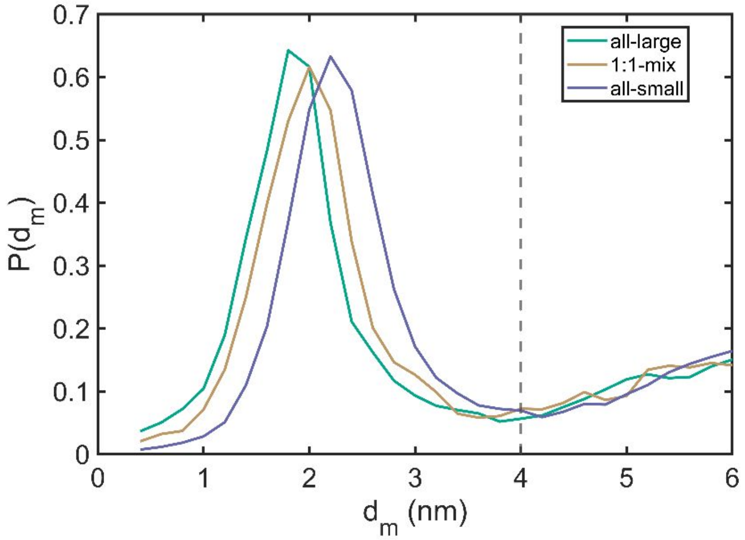

Figure S9. Probability distributions of the minimum distance between any two PQDs in coarse-grained MD simulations.

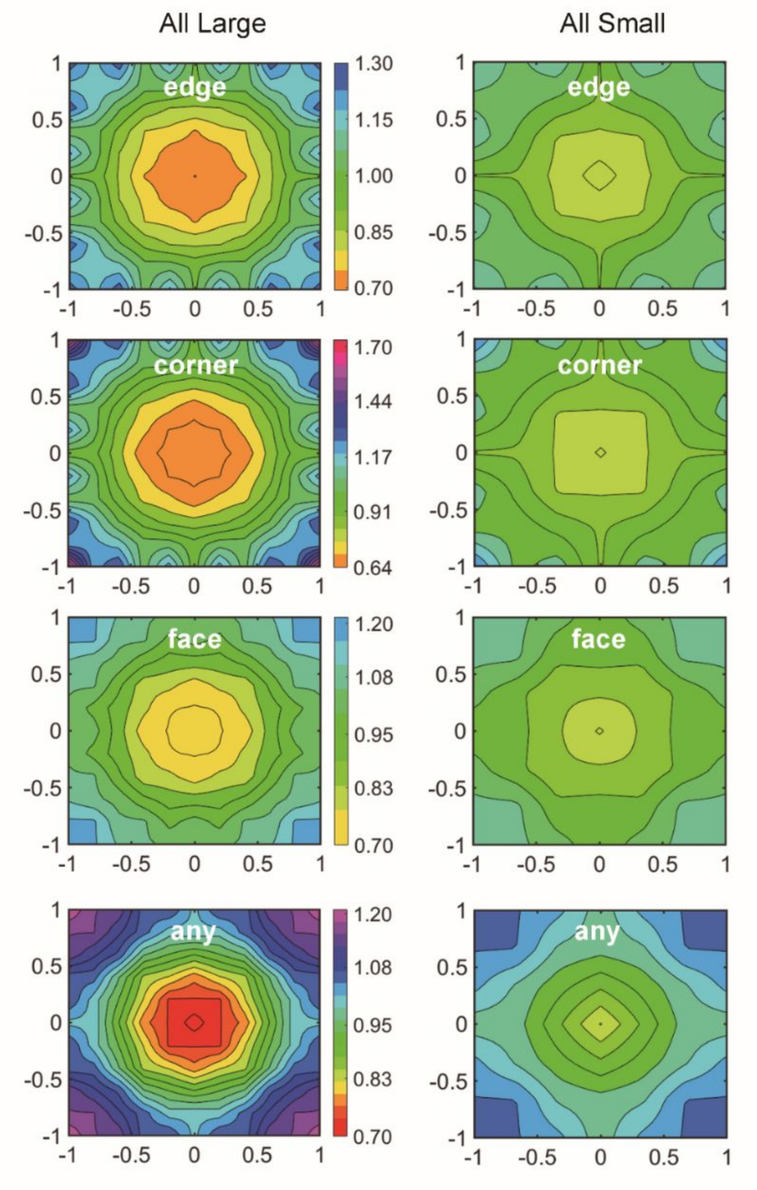

Figure S10. The contact maps of all-large (left column) and all-small (right column) PQD systems.

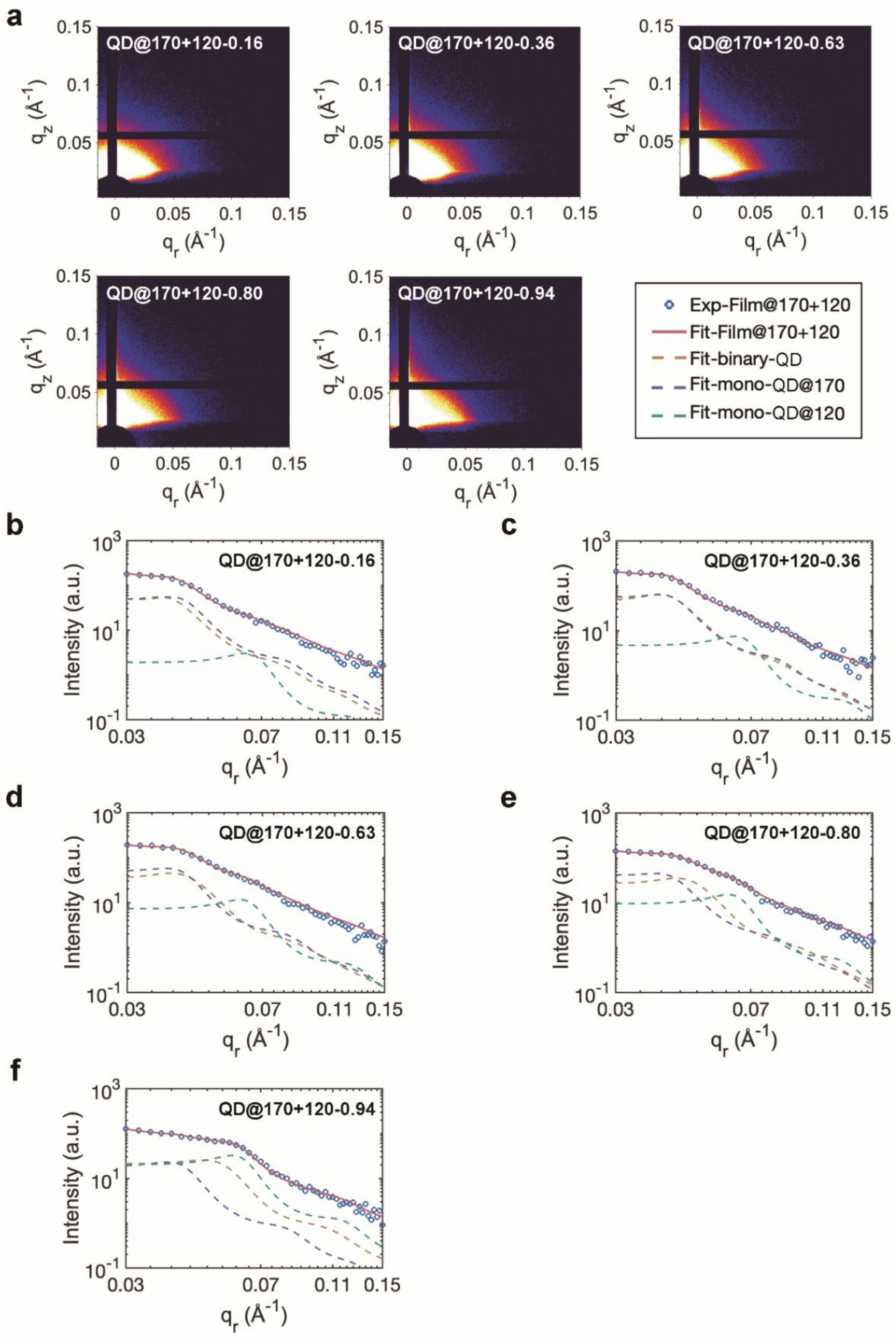

Figure S11. GISAXS measurement of PQDs after ligand exchange. (a) GISAXS 2D patterns of

ligand exchanged QD films with various number ratios; (b) to (f) are the linecut fitting of pure QD films and blend films with various number ratios after ligand exchange process. The samples are labelled with the film components and distinguished by the suffixes that correspond to the concentration of QD@120 in the film.

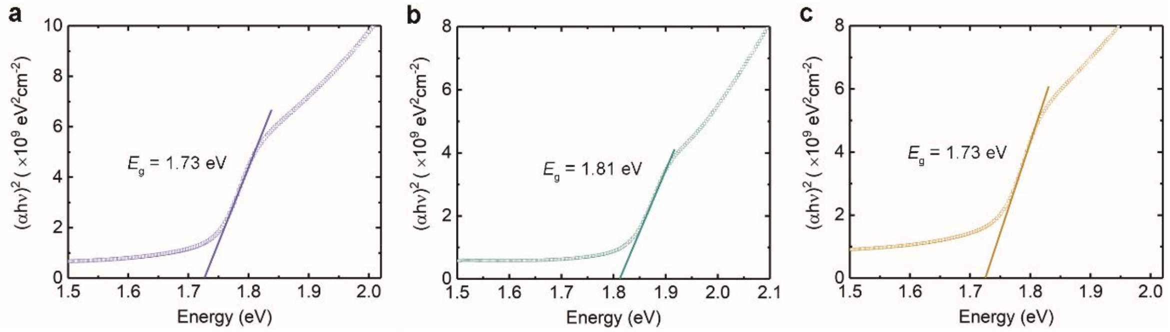

Figure S12. Tauc plot of absorption spectra. (a) QD@170, (b) QD@120, and (c) binary-sized QD@170+120-0.36 films.

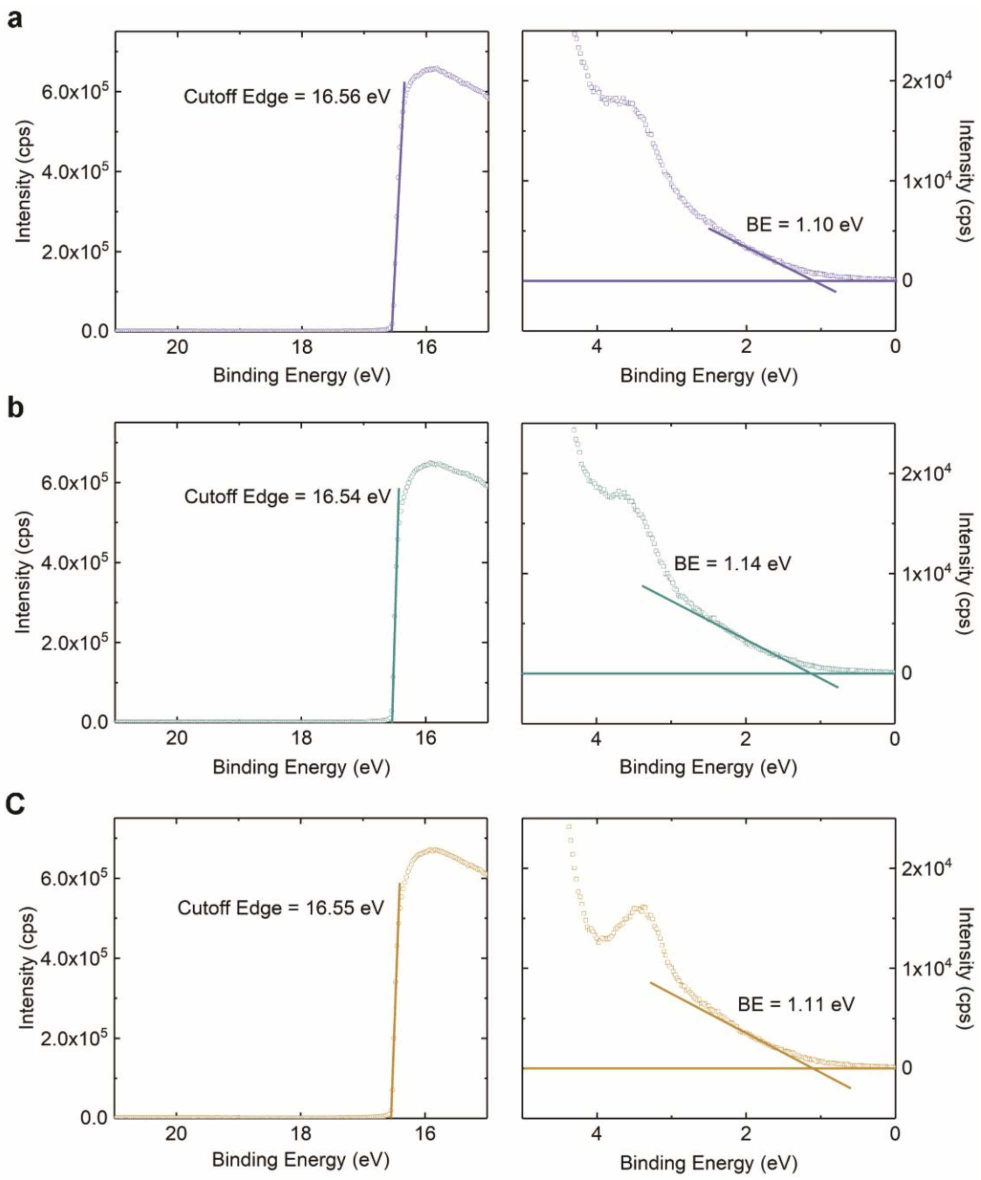

Figure S13. UPS spectra of (a) pure QD@170, (b) pure QD120, and (c) binary-sized QD@120-0.36 films.

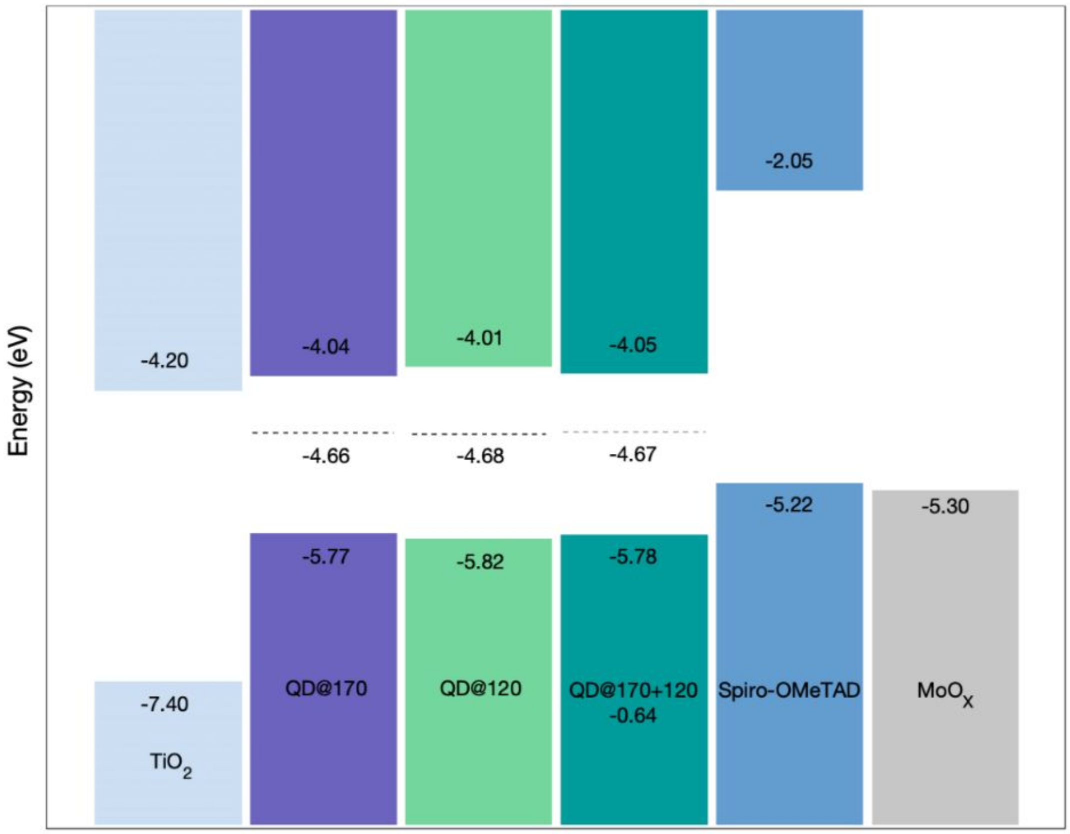

Figure S14 Band structure of assembled QD device

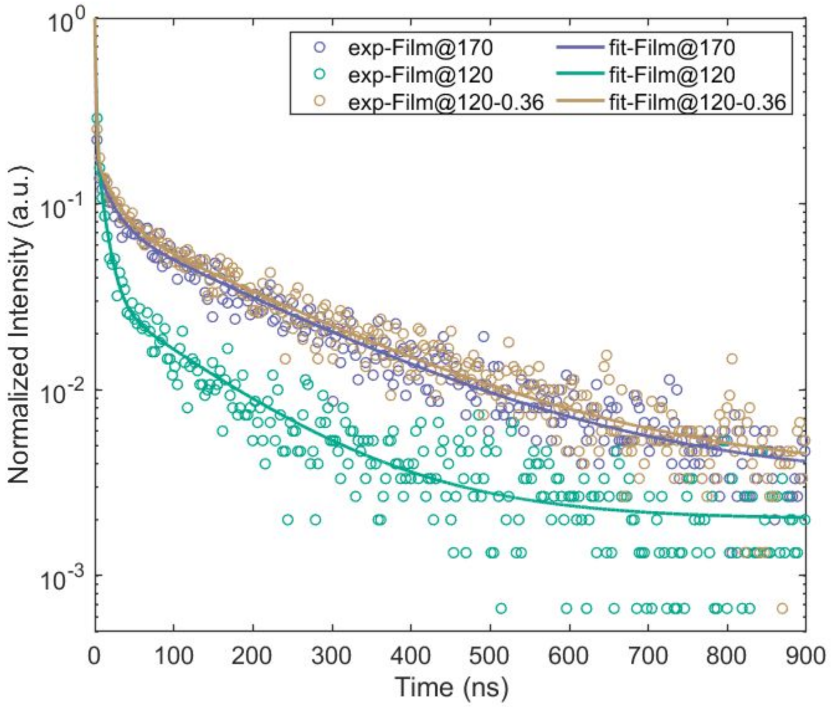

Figure S15 TRPL curves of QD films with varied number ratios. The curves are fitted with a tri-exponential function.

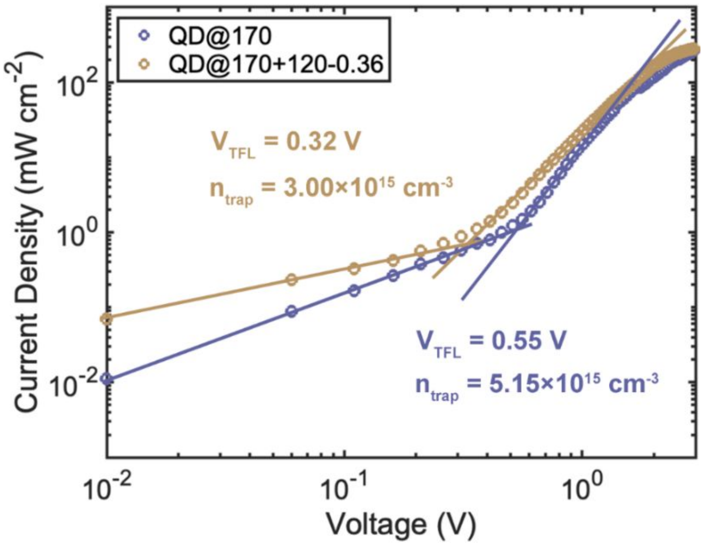

Figure S16 The space-charge-limited currents (SCLC) curves of PQD films fabricated by pure QD@170 and binary QD@170+120-0.36.

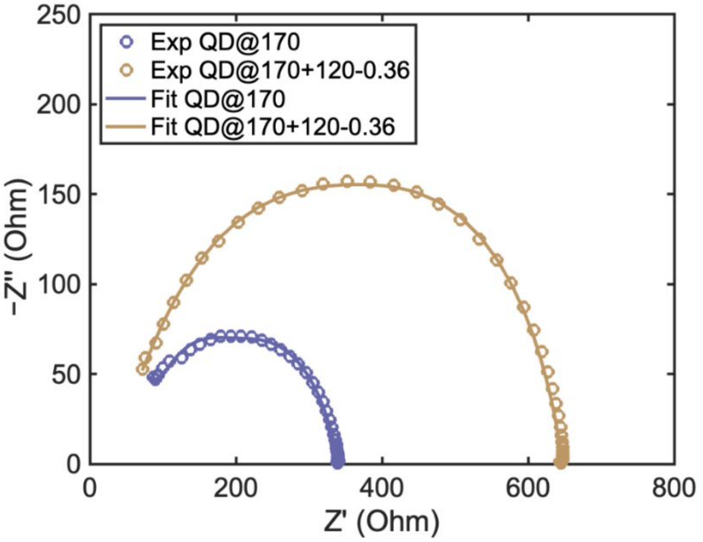

Figure S17 The EIS measurement of fabricated PQD device.

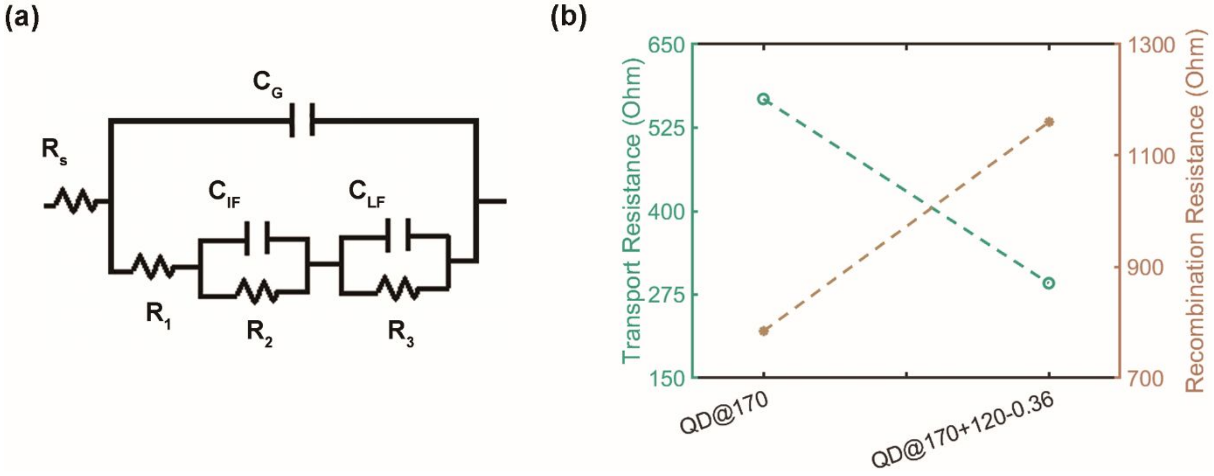

Figure S 18. (a) The equivalent circuit used to fit the EIS curves.[32] Rs is the series resistance originated by wires, connections and FTO substrate. C_G represents the geometric capacitance of the device at high frequency. The C_{IF}, C_{LF} represents the capacitance at intermediate and low frequency, respectively. It is hard to direct interpret the physical meaning of R_1, R_2 and R_3. However, the summation of all the R's gives the DC resistance, $R_{DC} = R_1+R_2+R_3$. The R_1+R_2 can provide the information about transport resistance[33, 34] in the selective contacts while the summation of $R_1+R_2+R_3$ can be considered as the recombination resistance[35, 36]. (b) Plotted the transport resistance and recombination resistance of the devices fabricated with pure QD@170 and binary film QD@170+120-0.36.

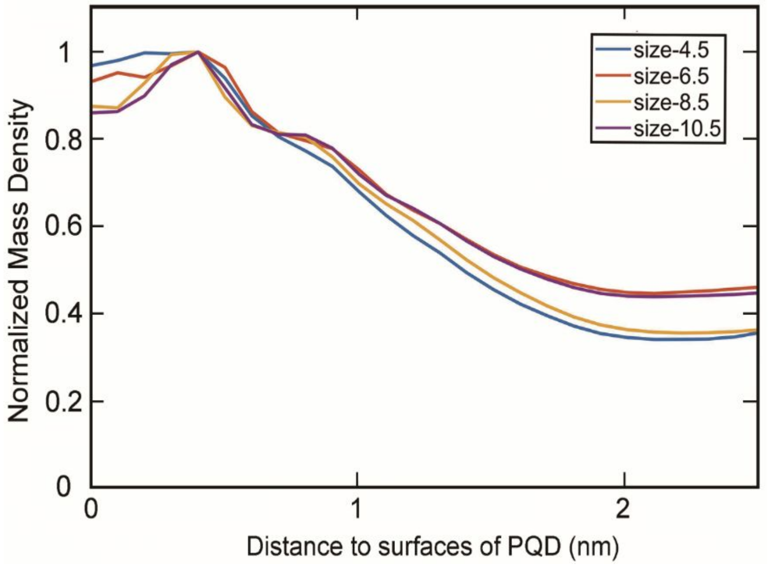

Figure S 19 Normalized mass density of head atoms of ligands within a given distance of a PQD surface obtained from all-atom MD simulations of various sized PQDs with long-chain ligands in octane.

Table S1 Fitted volume fraction η of mono-sized QD films

QD@170			QD@120		
**Size (nm)**	**Z value**	**Volume fraction η (%)**	**Size (nm)**	**Z value**	**Volume fraction η (%)**
12.6	21.8	34.7	8.68	27.1	35.7

Table S2 Ligand density of synthesized PQDs

QD type	Oleyl species (mM)	Ligand Density (nm^{-2})
**QD@170**	2.60	1.94
**QD@120**	3.35	2.38

Table S3 Fitted volume fraction η_1 of mono-sized QD films and total volume fraction η_2 under the consideration of particle softness. The ligands involved in calculation for softness is OA and OAm with the length of around 0.9 nm.

	QD diameter d (nm)	Fitted Volume fraction η_1 (%)	Softness λ	Total Volume fraction η_2 (%)	Z value
QD@170	12.6	34.7	0.143	51.9	21.8
QD@120	8.68	35.7	0.207	62.8	27.1

Table S4 Fitted volume fraction η of binary sized QD film before and after ligand exchanged.

Binary-component region		QD@170+120				
Concentration of QD@120		0.16	0.36	0.63	0.80	0.94
Fitted Volume fraction η (%)	Before Exchange	36.4	37.1	36.6	35.9	35.6
	After Exchange	37.0	37.8	37.1	36.4	36.1

Table S5 Averaged photovoltaic parameters of QD devices with champion data included in the bracket.

Concentration of QD@120	J_{SC} (mA cm^{-2})	V_{OC} (V)	Fill Factor (%)	Efficiency (%)
0	16.72 (17.02)	1.13 (1.11)	63.17 (67.45)	11.94 (12.75)
0.16	16.08 (16.10)	1.12 (1.16)	63.79 (65.01)	11.52 (12.18)

**0.36**	17.01 (17.08)	1.14 (1.19)	64.21 (71.12)	12.44 (14.42)
**0.63**	15.75 (15.82)	1.13 (1.19)	62.75 (65.16)	11.30 (12.28)
**0.80**	15.73 (15.85)	1.13 (1.17)	60.06 (60.46)	10.69 (11.23)
**0.94**	15.70 (15.75)	1.12 (1.15)	60.01 (60.22)	10.35 (10.83)
**1**	15.05 (15.52)	1.08 (1.10)	58.81 (61.38)	10.26 (10.72)

Table S6 TRPL fitted parameters of binary-sized QD film with varied concentration of QD@120.

**Concentration of QD@120**	A1	**τ_1 (ns)**	A2	**τ_2 (ns)**	**τ_{avg} (ns)**
**0**	0.91	1.23	0.09	20.32	13.07
**0.16**	0.94	1.26	0.09	23.33	15.37
**0.36**	0.9	1.34	0.1	24.53	16.89
**0.63**	0.92	1.28	0.1	21.64	14.47
**0.80**	0.86	1.25	0.11	19.75	13.63
**0.94**	0.92	1.19	0.12	17.92	12.28

where the average carrier lift time is calculated via the following formula[25]:

$$\tau_{avg} = \frac{A_1\tau_1^2 + A_2\tau_2^2}{A_1\tau_1 + A_2\tau_2} \qquad (26)$$

Appendix

$$A(\eta) = \frac{1 + 2\eta}{(1-\eta)^2} \quad B(\eta) = \frac{1 + \eta/2}{(1-\eta)^2} \tag{A1}$$

$$f_1(y) = \frac{y - sinsin\, y}{y^3} \quad f_2(y) = \frac{y - sinsin\, y}{y^3} \tag{A2}$$

$$f_3(y) = \frac{2f_2 + 1}{2y} \quad f_4(y) = -yf_1 \tag{A3}$$

$$a_1 = \frac{\partial}{\partial\eta_1}(\beta\rho') \quad a_2 = \alpha^{-3}\frac{\partial}{\partial\eta_2}(\beta\rho') \tag{A4}$$

where $(\beta\rho')$ is a reduced function

$$(\beta\rho') = \{(\eta_1 + \alpha^3\eta_2)(1 + \eta + \eta^2) - 3\eta_1\eta_2(1-\alpha)^2 \times [1 + \eta_1 + \alpha(1 + \eta_2)]\}(1-\eta)^{-3} \tag{A5}$$

$$2R_1b_1 = -6[\eta_1 g_{11}^2 + \tfrac{1}{4}\eta_2(1+\alpha)^2\alpha g_{12}^2] \tag{A6}$$

$$2R_2b_2 = -6[\eta_2 g_{22}^2 + \tfrac{1}{4}\eta_1(1+\alpha)^2\alpha^{-3} g_{12}^2] \tag{A7}$$

$$2R_2b = -3(1+\alpha)(\alpha^{-2}\eta_1 g_{11} + \eta_2 g_{22})g_{12} \tag{A8}$$

where

$$g_{11} = [\left(1 + \tfrac{1}{2}\eta\right) + \tfrac{3}{2}\eta_2(\alpha - 1)](1-\eta)^{-2} \tag{A9}$$

$$g_{22} = [\left(1 + \tfrac{1}{2}\eta\right) + \tfrac{3}{2}\eta_1(\tfrac{1}{\alpha} - 1)](1-\eta)^{-2} \tag{A10}$$

$$g_{12} = [\left(1 + \tfrac{1}{2}\eta\right) + \frac{31-\alpha}{21+\alpha}(\eta_1 - \eta_2)](1-\eta)^{-2} \tag{A11}$$

$$(2R_1)^3 d = (\eta_1 a_1 - \alpha^3 \eta_2 a_2) \quad \text{(A12)}$$

The total scattering intensity can be termed as

$$I \propto xS_{22}P_2 + 2[x(1-x)]^{1/2} S_{12}(P_1P_2)^{1/2} + (1-x)S_{11}P_2 \quad \text{(A13)}$$

REFERENCES

(1) Lu, X.; Mochrie, S. G.; Narayanan, S.; Sandy, A. R.; Sprung, M. How a liquid becomes a glass both on cooling and on heating. *Phys Rev Lett* **2008**, *100* (4), 045701.
(2) Lu, X.; Mochrie, S. G. J.; Narayanan, S.; Sandy, A. R.; Sprung, M. Temperature-dependent structural arrest of silica colloids in a water–lutidine binary mixture. *Soft Matter* **2010**, *6* (24), 6160-6177.
(3) Weir, M. P.; Toolan, D. T. W.; Kilbride, R. C.; Penfold, N. J. W.; Washington, A. L.; King, S. M.; Xiao, J.; Zhang, Z.; Gray, V.; Dowland, S.; et al. Ligand Shell Structure in Lead Sulfide-Oleic Acid Colloidal Quantum Dots Revealed by Small-Angle Scattering. *J Phys Chem Lett* **2019**, *10* (16), 4713-4719.
(4) Griffith, W. L.; Triolo, R.; Compere, A. L. Analytical scattering function of a polydisperse Percus-Yevick fluid with Schulz-(Gamma-) distributed diameters. *Phys Rev A Gen Phys* **1987**, *35* (5), 2200-2206.
(5) Michael Kotlarchyk, R. B. S., 1 and John S. Huang*1. Study of Schultz Distribution to Model Polydlsperslty of Microemulsion Droplets. *The Journal of Physical Chemistry* **1988**, *92* (6), 1533-1538.
(6) Torquato, S. Hyperuniform states of matter. *Physics Reports* **2018**, *745*, 1-95.
(7) Nägele, G. *The Physics of Colloidal Soft Matter*; Institute of Fundamental Technological Research Świętokrzyska 21, 00-049 Warszawa, 2004.
(8) Lebowitz, J. L. Exact Solution of Generalized Percus-Yevick Equation for a Mixture of Hard Spheres. *Physical Review* **1964**, *133* (4A), A895-A899.
(9) Ashcroft, N. W.; Langreth, D. C. Structure of Binary Liquid Mixtures. I. *Physical Review* **1967**, *156* (3), 685-692.
(10) Li, X.; Hoffman, J. M.; Kanatzidis, M. G. The 2D Halide Perovskite Rulebook: How the Spacer Influences Everything from the Structure to Optoelectronic Device Efficiency. *Chem Rev* **2021**, *121* (4), 2230-2291.
(11) Mao, L.; Stoumpos, C. C.; Kanatzidis, M. G. Two-Dimensional Hybrid Halide Perovskites: Principles and Promises. *J Am Chem Soc* **2019**, *141* (3), 1171-1190.
(12) Stelmakh, A.; Aebli, M.; Baumketner, A.; Kovalenko, M. V. On the Mechanism of Alkylammonium Ligands Binding to the Surface of CsPbBr3 Nanocrystals. *Chem Mater* **2021**, *33* (15), 5962-5973.
(13) ten Brinck, S.; Infante, I. Surface Termination, Morphology, and Bright Photoluminescence of Cesium Lead Halide Perovskite Nanocrystals. *ACS Energy Letters* **2016**, *1* (6), 1266-1272.
(14) Best, R. B.; Zhu, X.; Shim, J.; Lopes, P. E.; Mittal, J.; Feig, M.; Mackerell, A. D., Jr. Optimization of the additive CHARMM all-atom protein force field targeting improved sampling of the backbone phi, psi and side-chain chi(1) and chi(2) dihedral angles. *J Chem Theory Comput* **2012**, *8* (9), 3257-3273.
(15) Vanommeslaeghe, K.; MacKerell, A. D., Jr. Automation of the CHARMM General Force Field (CGenFF) I: bond perception and atom typing. *J Chem Inf Model* **2012**, *52* (12), 3144-3154.
(16) Vanommeslaeghe, K.; Raman, E. P.; MacKerell, A. D., Jr. Automation of the CHARMM General Force Field (CGenFF) II: assignment of bonded parameters and partial atomic charges. *J Chem Inf Model* **2012**, *52* (12), 3155-3168.
(17) Jo, S.; Kim, T.; Iyer, V. G.; Im, W. CHARMM-GUI: a web-based graphical user interface for CHARMM. *J Comput Chem* **2008**, *29* (11), 1859-1865.
(18) Vanommeslaeghe, K.; Hatcher, E.; Acharya, C.; Kundu, S.; Zhong, S.; Shim, J.; Darian, E.;

Guvench, O.; Lopes, P.; Vorobyov, I.; et al. CHARMM general force field: A force field for drug-like molecules compatible with the CHARMM all-atom additive biological force fields. *J Comput Chem* **2010**, *31* (4), 671-690.
(19) Yu, W.; He, X.; Vanommeslaeghe, K.; MacKerell, A. D., Jr. Extension of the CHARMM General Force Field to sulfonyl-containing compounds and its utility in biomolecular simulations. *J Comput Chem* **2012**, *33* (31), 2451-2468.
(20) Mayne, C. G.; Saam, J.; Schulten, K.; Tajkhorshid, E.; Gumbart, J. C. Rapid parameterization of small molecules using the Force Field Toolkit. *J Comput Chem* **2013**, *34* (32), 2757-2770.
(21) *Gaussian 09, Revision A.02*; Gaussian, Inc., Wallingford CT: 2016. (accessed.
(22) In Suk Joung, T. E. C., III. Determination of Alkali and Halide Monovalent Ion Parameters for Use in Explicitly Solvated Biomolecular Simulations. *J. Phys. Chem. B* **2008**, *112*, 9020–9041.
(23) Li, P.; Roberts, B. P.; Chakravorty, D. K.; Merz, K. M., Jr. Rational Design of Particle Mesh Ewald Compatible Lennard-Jones Parameters for +2 Metal Cations in Explicit Solvent. *J Chem Theory Comput* **2013**, *9* (6), 2733-2748.
(24) Bussi, G.; Donadio, D.; Parrinello, M. Canonical sampling through velocity rescaling. *J Chem Phys* **2007**, *126* (1), 014101.
(25) Abraham, M. J.; Murtola, T.; Schulz, R.; Páll, S.; Smith, J. C.; Hess, B.; Lindahl, E. GROMACS: High performance molecular simulations through multi-level parallelism from laptops to supercomputers. *SoftwareX* **2015**, *1-2*, 19-25.
(26) William Humphrey, A. D., Klaus Schulten. VMD: Visual Molecular Dynamics. *Journal of Molecular Graphics* **1996**, *14*, 33-38.
(27) Aksimentiev, A.; Schulten, K. Imaging alpha-hemolysin with molecular dynamics: ionic conductance, osmotic permeability, and the electrostatic potential map. *Biophys J* **2005**, *88* (6), 3745-3761.
(28) K. Chen; W. Jin; Y. Zhang; T. Yang; P. Reiss; Q. Zhong; U. Bach; Q. Li; Y. Wang; H. Zhang; et al. High Efficiency Mesoscopic Solar Cells Using CsPbI3 Perovskite Quantum Dots Enabled by Chemical Interface Engineering. *J Am Chem Soc* **2020**, *142* (8), 3775-3783.
(29) Li, J.; Xu, L.; Wang, T.; Song, J.; Chen, J.; Xue, J.; Dong, Y.; Cai, B.; Shan, Q.; Han, B.; et al. 50-Fold EQE Improvement up to 6.27% of Solution-Processed All-Inorganic Perovskite CsPbBr3 QLEDs via Surface Ligand Density Control. *Adv Mater* **2017**, *29* (5).
(30) Cherniukh, I.; Rainò, G.; Stöferle, T.; Burian, M.; Travesset, A.; Naumenko, D.; Amenitsch, H.; Erni, R.; Mahrt, R. F.; Bodnarchuk, M. I.; et al. Perovskite-type superlattices from lead halide perovskite nanocubes. *Nature* **2021**, *593* (7860), 535-542.
(31) X. Z. An; S. S. He; H. D. Feng; Q. Qian. Packing densification of binary mixtures of spheres and cubes subjected to 3D mechanical vibrations. *Applied Physics A* **2014**, *118* (1), 151-162.
(32) Zolfaghari, Z.; Hassanabadi, E.; Pitarch-Tena, D.; Yoon, S. J.; Shariatinia, Z.; van de Lagemaat, J.; Luther, J. M.; Mora-Seró, I. Operation Mechanism of Perovskite Quantum Dot Solar Cells Probed by Impedance Spectroscopy. *ACS Energy Letters* **2018**, *4* (1), 251-258.
(33) Guerrero, A.; Garcia-Belmonte, G.; Mora-Sero, I.; Bisquert, J.; Kang, Y. S.; Jacobsson, T. J.; Correa-Baena, J.-P.; Hagfeldt, A. Properties of Contact and Bulk Impedances in Hybrid Lead Halide Perovskite Solar Cells Including Inductive Loop Elements. *The Journal of Physical Chemistry C* **2016**, *120* (15), 8023-8032.
(34) Juarez-Perez, E. J.; Wubetaler, M.; Fabregat-Santiago, F.; Lakus-Wollny, K.; Mankel, E.; Mayer, T.; Jaegermann, W.; Mora-Sero, I. Role of the Selective Contacts in the Performance of Lead Halide Perovskite Solar Cells. *J Phys Chem Lett* **2014**, *5* (4), 680-685.
(35) Zarazua, I.; Han, G.; Boix, P. P.; Mhaisalkar, S.; Fabregat-Santiago, F.; Mora-Sero, I.;

Bisquert, J.; Garcia-Belmonte, G. Surface Recombination and Collection Efficiency in Perovskite Solar Cells from Impedance Analysis. *J Phys Chem Lett* **2016**, *7* (24), 5105-5113.
(36) Zarazua, I.; Sidhik, S.; Lopez-Luke, T.; Esparza, D.; De la Rosa, E.; Reyes-Gomez, J.; Mora-Sero, I.; Garcia-Belmonte, G. Operating Mechanisms of Mesoscopic Perovskite Solar Cells through Impedance Spectroscopy and J-V Modeling. *J Phys Chem Lett* **2017**, *8* (24), 6073-6079.
